# Single-Cell Metabolic Profiling of Macrophages Using 3D OrbiSIMS: Correlations with Phenotype

**DOI:** 10.1021/acs.analchem.2c01375

**Published:** 2022-06-17

**Authors:** Waraporn Suvannapruk, Max K. Edney, Dong-Hyun Kim, David J. Scurr, Amir M. Ghaemmaghami, Morgan R. Alexander

**Affiliations:** † Advanced Materials and Healthcare Technologies Division, School of Pharmacy, University of Nottingham, University Park, Nottingham NG7 2RD, United Kingdom; ‡ Department of Chemical and Environmental Engineering, Faculty of Engineering, University of Nottingham, University Park, Nottingham NG7 2RD, United Kingdom; § Immunology & Immuno-bioengineering Group, School of Life Sciences, Faculty of Medicine and Health Sciences, University of Nottingham, University Park, Nottingham NG7 2RD, United Kingdom

## Abstract

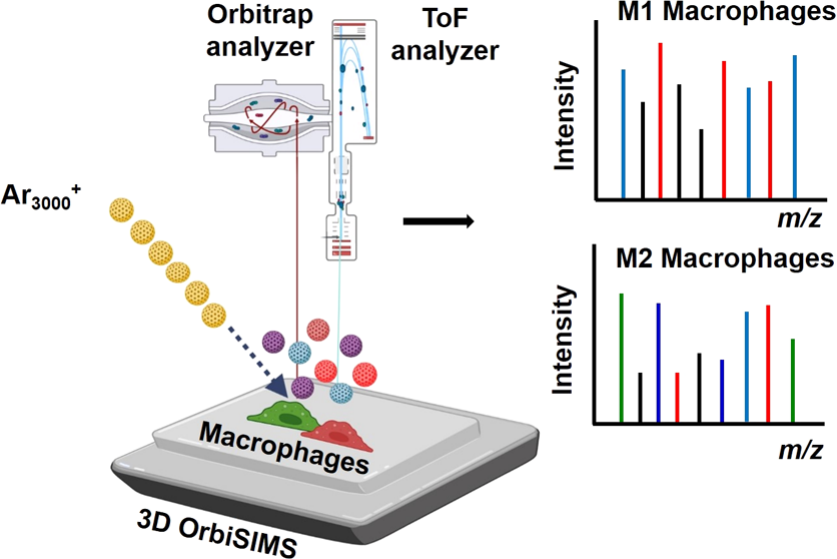

Macrophages are important immune cells that respond to environmental cues acquiring a range of activation statuses represented by pro-inflammatory (M1) and anti-inflammatory (M2) phenotypes at each end of their spectrum. Characterizing the metabolic signature (metabolic profiling) of different macrophage subsets is a powerful tool to understand the response of the human immune system to different stimuli. Here, the recently developed 3D OrbiSIMS instrument is applied to yield useful insight into the metabolome from individual cells after in vitro differentiation of macrophages into naïve, M1, and M2 phenotypes using different cytokines. This analysis strategy not only requires more than 6 orders of magnitude less sample than traditional mass spectrometry approaches but also allows the study of cell-to-cell variance. Characteristic metabolites in macrophage subsets are identified using a targeted lipid and data-driven multivariate approach highlighting amino acids and other small molecules. The diamino acids alanylasparagine and lipid sphingomyelin SM(d18/16:0) are uniquely found in M1 macrophages, while pyridine and pyrimidine are observed at increased intensity in M2 macrophages, findings which link to known biological pathways. The first demonstration of this capability illustrates the great potential of direct cell analysis for in situ metabolite profiling with the 3D OrbiSIMS to probe functional phenotype at the single-cell level using molecular signatures and to understand the response of the human body to implanted devices and immune diseases.

## Introduction

Macrophages are the sentinels and regulators of the human immune system; they display remarkable stimulus-induced functional plasticity, which is key in their ability in responding to a diverse range of pathogens, foreign objects, and dead cells resulting from tissue injury. Following infection or tissue damage, macrophages acquire a spectrum of functional phenotypes that are exemplified by pro-inflammatory (M1) and anti-inflammatory (M2) phenotypes at each end of the spectrum, with these subsets expressing unique biomarkers.^1^ In vitro, classically activated M1 macrophages are generated by polarizing monocyte-derived naïve macrophages (M0) with a mix of cytokines including interferon γ and granulocyte macrophage colony-stimulating factor (GM-CSF).^2,3^ M1-like macrophages play an important role in pathogen clearance, and their sustained activation is linked to postinjury tissue damage. As indicated by their name, they secrete high levels of pro-inflammatory cytokines such as TNF-α, IL-12, and IL-1β, and metabolically they are known to turn on glycolysis, the pentose phosphate pathway, and fatty acid synthesis.^4−7^ M2 macrophages on the other hand can be polarized using interleukin-4 (IL-4) and macrophage colony-stimulating factor (M-CSF)^8,9^ and have been implicated in tissue repair and remodeling; however, their sustained activation has been linked to the promotion of fibrosis. M2-like macrophages typically produce high levels of regulatory cytokines such as IL-10 and TGF-β and, unlike M1 macrophages, rely on the TCA cycle to support oxidative phosphorylation and fatty acid oxidation which generate ATP.^10−12^

The traditional approach for phenotyping macrophages relies on quantifying their transcription factors, cytokine profile, or expression of surface markers for different subsets. M1 macrophages had the highest expression of calprotectin and produced high levels of TNF-α, whereas M2 macrophages have a higher expression of mannose receptor (CD206) and produce high levels of IL-10.^13−16^ However, this is problematic since some of these markers are expressed by both M1 and M2 macrophage types, and importantly many, especially surface markers do not provide meaningful insight into macrophage functional properties.^17,18^

Metabolomics is a powerful analytical tool^19^ that shows promise for distinguishing between macrophage subsets by identifying subtle yet key differences between their metabolic profiles. Metabolomics was developed to investigate the metabolic state of cells that closely correlates with their instantaneous functional state, including their response to stimuli from their environment.^20^ There are a wide range of techniques for metabolic analysis including mass spectrometry. Liquid chromatography–mass spectrometry (LC-MS) is a powerful tool for quantifying a range of small biomolecules and identifying known and unknown metabolites present in biological samples. This has been employed widely for identifying biomarkers of inflammatory diseases, drug discovery, and in the study of cellular metabolic profiles.^21−24^ Despite recent advances in determining the metabolic phenotype of various pathologies using conventional LC-MS-based techniques,^25−27^ characterizing the metabolome of different cells that are thought to play an important role in the development of such pathologies (e.g., macrophage subsets) is still in its formative stages partly due to the need for a large number of cells (>1 million cells) to obtain adequate signal.^28,29^ Furthermore, LC-MS-based techniques require complex and extensive sample preparation procedures including metabolic quenching and extraction of intracellular metabolites from cells using organic solvents. These procedures that target one metabolite type can lead to incomplete sampling and biased quantification of small molecules in biological samples due to rapid turnover and loss of metabolites.^1,28,30^ Moreover, cell samples are homogenized (metabolite extraction) during the sample preparation, thus losing the architecturally intact and physiologically relevant structure of the tissue and cells and eliminating the potential to correlate the spatial distribution of metabolites to morphology. Time-of-flight secondary ion MS (TOF-SIMS) is a direct surface analysis technique with minimal sample preparation, which can perform chemical imaging and depth profiling of cells.^31,32^ Previous attempts at metabolic profiling using this technique targeted key molecules such as lipids and cholesterol on the mast cell.^33^ Touboul et al. applied TOF-SIMS imaging to study metabolites and mechanisms of disease on the tissue sample.^34^ The development of TOF-SIMS with polyatomic primary beams has provided the benefits of high molecular yields and low subsurface damage.^35^ But TOF-SIMS has been limited to resolving and detecting metabolites.^36^ However, TOF-SIMS has not been used widely for metabolite profiling of cells because its poor mass resolving power hinders confident identification of endogenous metabolites.^37^

The 3D OrbiSIMS is a recently developed technique bringing together the state-of-the-art Orbitrap analyzer with TOF-SIMS.^38^ 3D OrbiSIMS combines the strengths of a TOF analyzer with its fast imaging acquisition time and high spatial resolution (<200 nm), the high mass resolving power (>240,000) of the OrbitrapTM (parts-per-million (ppm) (<2)) and mass accuracy, high sensitivity, and tandem MS (MS/MS) capability. The technique uses an argon gas cluster primary ion beam (GCIB), affording a low energy per atom (*E*/*n*), and has been shown to liberate and unambiguously identify large diagnostic chemical species such as lipids and peptides.^38,39^ Passarelli et al. have used this novel technique for metabolite profiling of macrophages treated with different concentrations of an exogenous compound, the drug amiodarone, imaging it with endogenous compounds in single cells. In a tissue section of mouse brain, they identified lipid and amino acid fragments and were able to image a single cell in a tissue section.^38^ Hodgkinson et al. have recently used 3D OrbiSIMS images to observe metabolites in multiple mesenchymal stem cells (MSCs) using ToF and Orbitrap.^40^

Single-cell SIMS analysis represents an exciting method for single-cell metabolomic profiling to probe intercell variations. Here, we use 3D OrbiSIMS to investigate the metabolic profiles of human monocyte-derived M0, M1, and M2 macrophages at single-cell level, leading to identifying characteristic ions that are related to known biological processes for each subset.

## Experimental Section

### Sample Preparation

Buffy coats from healthy donors were collected from the National Blood Service (National Blood Service, Sheffield, U.K.), following ethics committee approval (2009/D055, Research Ethics Committee, Faculty of Medicine and Health Sciences, University of Nottingham). Peripheral blood mononuclear cells (PBMCs) were isolated from heparinized blood by Histopaque-1077 (Sigma-Aldrich) density gradient centrifugation, as previously described.^15^ A total of 2 × 10^5^ monocytes were seeded on indium tin oxide (ITO) glass slides and cultured in RPMI supplement with 10% fetal bovine serum (FBS), 10 μg/mL streptomycin, 2 mM L-glutamine, and 10 U/mL penicillin. Monocytes M0 were differentiated into M1 and M2 macrophages by the addition of 20 ng/mL M-CSF, 50 ng/mL GM-CSF, and 20 ng/mL IFN-α (R&D Systems), 50 ng/mL M-CSF, and 20 ng/mL IL-4, respectively, as described previously.^41^ All cytokines were from Miltenyi Biotec unless otherwise stated (Figure S1). The cells were incubated in a 37 °C incubator in a humidified atmosphere of 5% CO_2_ for 6 days. On day 3 of incubation, we replaced 500 μL of medium with fresh media supplemented with the same concentration and mix of cytokines that were used for cell stimulation on day 0. Cell supernatant was harvested on day 6 for cytokine analysis, and cells were collected for analysis.

### Analysis of Macrophage Surface Phenotype

**Figure 1:**
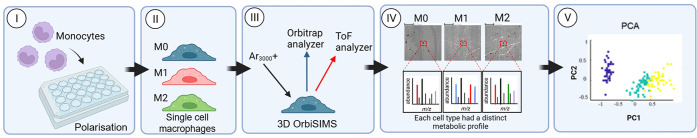
Schematic workflow of single-cell metabolomic profiling using 3D OrbiSIMS. Here, monocytes cells M0 are grown on an ITO substrate and polarized toward M1 and M2 macrophages. A gas cluster argon primary ion beam raster is used to identify and sputter single cells with the resultant complex spectra compared using multivariate analysis complemented with targeted analysis.

This was carried out as previously described with some modifications.^15^ Briefly, on day 6, the cells were fixed in 4% paraformaldehyde in PBS for 10 min at room temperature (RT). This was followed by washing cells with 3% bovine serum albumin (BSA) and 1% glycine (Fischer Scientific) in PBS. The cells were then incubated with appropriately diluted primary antibodies; 2 μg/mL mouse anti-human calprotectin (27E10) (Thermo Scientific) and 1 μg/mL rabbit anti-human MR (CD206) (Abcam) in 5% goat serum (GS) for 1 h followed by three times washing with PBS and addition of appropriately diluted secondary antibodies; and 8 μg/mL Rhodamine Red-x goat anti-mouse IgG(H + L) (Life Technologies) and 8 μg/mL Alexa Fluor 488 goat anti-rabbit (H+L) in 5% goat serum (GS) and further 1 h incubation at RT in the dark followed by three times washing with PBS. The nuclei of the cells were stained with 4′,6-diamidino-2-phenylindole (DAPI, 20,000 ng/mL) for 5 min at RT in the dark. The cells were washed, dried, and finally mounted onto a slide with mounting media (Prolong antifade kit). The stained cells were imaged using ZOE fluorescence cell imager. Images were analyzed using CellProfiler Cell Image Analysis Software, and fluorescence images of the expression of MR and calprotectin marker were analyzed to determine the intensity of MR and calprotectin. The full method is depicted as a schematic in Figure 1.

### Cytokine Quantification

On day 6, supernatants were collected for cytokine assay. The levels of IL-10 and TNF-α in the culture media were measured by DuoSet ELISA kit (R&D Systems) following manufacturer’s instructions.

### 3D OrbiSIMS Analysis

To remove salts that cause unwanted signal suppression in SIMS, the cells on ITO slides from the cell culture experiments were put in a container and then 1 mL of 150 mM ammonium formate solution was added for 30 s three times. The sample was plunge-frozen in liquid nitrogen and freeze-dried over a period of 12 h to remove water. The sample was subsequently stored in a sealed container and stored at −80 °C until analysis. Prior to OrbiSIMS analysis, the sample was warmed to room temperature without opening and then loaded into the 3D OrbiSIMS instrument airlock for analysis.

3D OrbiSIMS analysis was performed using a HybridSIMS instrument (IONTOF, GmbH) with Mode 4 (single beam 20 keV Ar_3000_^+^, OrbitrapTM analyzer) of the instrument^38^ using an Ar_3000_^+^ primary ion beam of energy 20 keV, a duty cycle of 4.4%, and continuous GCIB current of 230 pA over an area of 150 × 150 μm^2^ with crater size 233.1 × 233.1 μm^2^ in the mass range of *m*/*z* 75–1125. The electron flood gun was operated with an energy of 21 eV and an extraction bias of 20 V for charge compensation. The pressure in the main chamber was maintained at 8.2 × 10^–8^ mbar. The OrbitrapTM cycle time was set to 200 μs. The OrbitrapTM analyzer was operated in positive- and negative-ion modes at the 240,000 at *m*/*z* 200 mass resolution setting. The injection time was 500 ms, and the total ion dose per measurement was 3.95 × 10^11^ ions/cm^2^. Adjacent areas on the cell were analyzed for positive and negative polarities. In each case, an entire cell was consumed during each polarity analysis. Eighteen cells were consumed in total (3 cells per type and 1 cell per polarity) (Figure S2).

### Principal Component Analysis (PCA)

3D OrbiSIMS spectra contained hundreds of ions, and the mass spectra of samples superficially appear similar to each other. Depth profile accumulation spectra of all single cells analyzed for M0, M1, and M2 macrophages (three cells of each type, nine in total) were each normalized to their respective total ion count in SurfaceLab 7 software. A peak list containing the intense ions (minimum ion count threshold was determined in each case as being greater than assigned noise signals) was then constructed with normalized intensities. A common peak list containing each ion for all samples was then constructed in SurfaceLab 7 software, which contained 724 positive ions. We performed PCA on this dataset to summarize differences in sample chemistry using PCA Bundle software.

### Data Processing and Metabolites Identification

Peak assignments were created by IonTOF SurfaceLab 7. Amino acid fragments were assigned using an Xcalibur to create the peak lists of each cell type. 3D OrbiSIMS spectra were exported as .txt files. Metabolites results were searched against the Human Metabolome Database^42^ with 5 ppm mass tolerance for putative annotation.

## Results and Discussion

### Characterization of Macrophage Marker Expression and Cytokines Analysis

**Figure 2:**
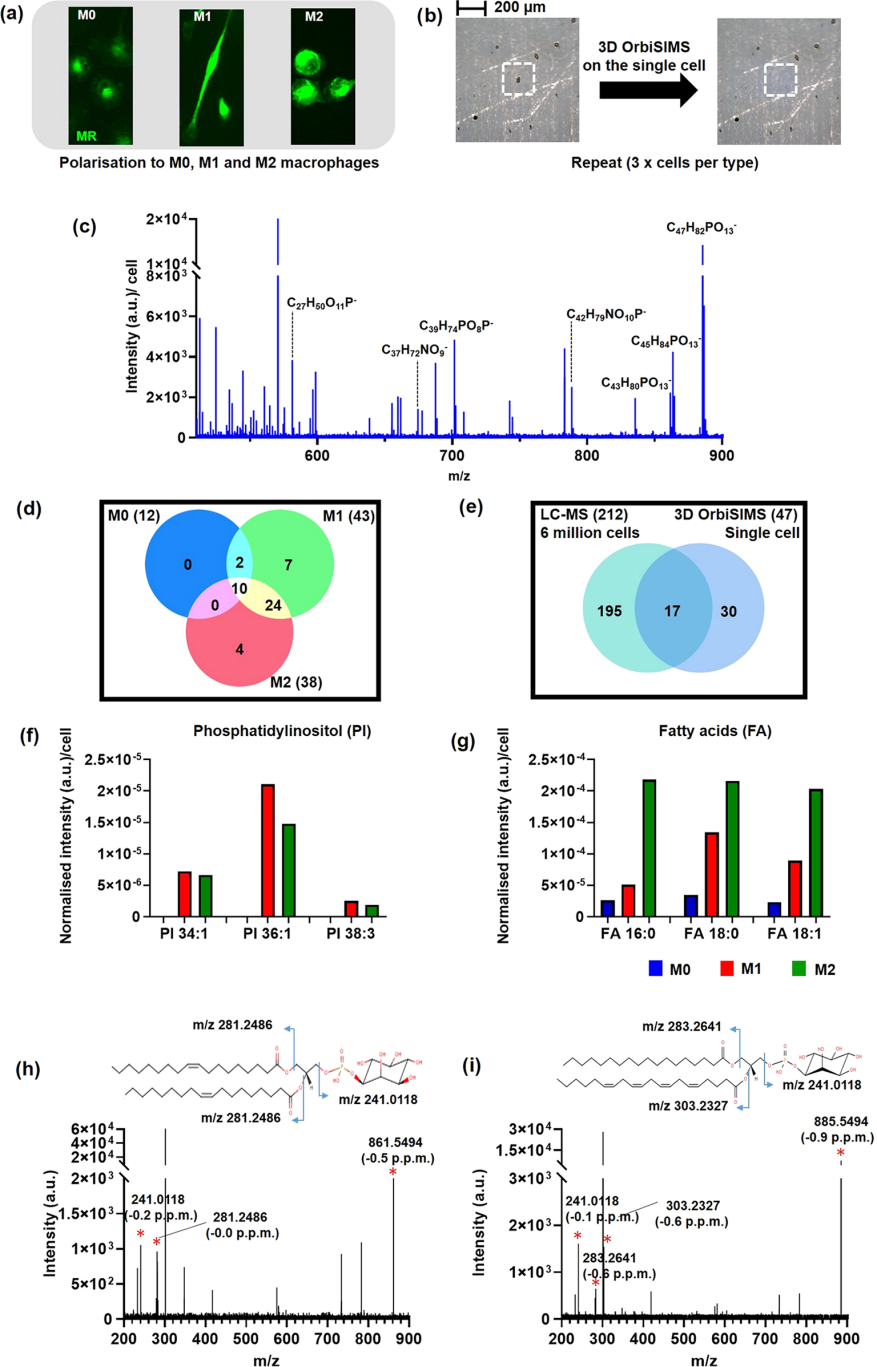
(a) Fluorescent images of single-cell macrophages stained for mannose receptor. (b) White image of the M0 cell type before (left) and after (right) 3D OrbiSIMS depth profiling, showing consumption of the cell by the gas cluster ion beam. Targeted metabolomics approach of assigning lipid species in 3D OrbiSIMS data and comparison to LC-MS data from ref (28). (c) Negative-ion mass spectrum 3D OrbiSIMS of the lipid fragments from a single-cell macrophage. (d) Venn diagram comparison of the number of lipid compounds detected in macrophages subsets using 3D OrbiSIMS. (e) Venn diagram comparing the number of lipid compounds in macrophages by LC-MS and 3D OrbiSIMS measurement; 212 lipids were identified in THP-1 macrophages extracts using LC-MS,^28^ 47 lipids were identified by 3D OrbiSIMS, and 17 lipid compounds were common to both techniques. Normalized intensity of lipid classes with characteristic ion data shown for (f) phosphatidylinositol species (PI) and (g) fatty acids. Species of phosphatidylinositol (PI) confirmation by MS/MS. (h) MS/MS product spectrum of PI (36:2) corresponding to [M – H]^−^ ion at *m*/*z* 861.5494. (i) MS/MS product spectrum of PI (38:4) corresponding to [M – H]^−^ ion at *m*/*z* 885.5498.

Before analysis with 3D OrbiSIMS, we first polarized monocyte cells (M0) into different macrophage phenotypes (M1 and M2) shown in Figure 2a. The surface phenotype and cytokine profile of each subset were characterized using optical microscopy and ELISA respectively. Data presented in Figure S1 show that M1 macrophages had the highest expression of calprotectin and produced high levels of TNF-α, whereas M2 macrophages have a higher expression of mannose receptor (CD206) and produce high levels of IL-10. These are in line with the expected phenotype of these cells that we and others have previously shown.^43−46^

### Targeted Lipid Analysis

We performed 3D OrbiSIMS depth profile analysis on single cells of each type, where the cells were sequentially consumed by sputtering using the Ar3000+ gas cluster ion beam. The optical images of single cells acquired within the instrument before and after analysis by the gas cluster ion beam are shown in Figure 2b. Peak lists from the OrbiTrap analysis of the cells were first analyzed using a targeted approach by matching peaks with ions of lipid species from the LIPID MAPS database based on their mass.^47^ A representative negative mass spectrum of lipids from a single-macrophage cell is presented in Figure 2c. The number of lipid species found in each macrophage subset and common to each are summarized in Figure 2d. A greater number of lipid compounds were detected in M1 macrophages than in M0 and M2. The identity of these presented in Table S1 and Table S5, including phosphatidylinositol lipid species such as PI 34:1, PI 36:1, and PI 38:3, which were highest in M1, while PI 34:1, PI 36:1, and PI 38:3 were not detected in M0 (Figure 2f and Table S1). Free fatty acid species, FA 16:0, FA 18:0, and FA 18:1 were lowest in M0 and highest in M2 samples (Figure 2g and Table S1). M1 polarization is associated with the activation of fatty acid synthesis. While M2 macrophages are known to have increased fatty acid oxidation and enhanced metabolism and upregulated activities that are associated with tissue remodeling or wound healing.^48^ Passarelli^38^ studied lipid compounds in tissue section by 3D OrbiSIMS and using the LIPID MAPS database putatively annotate 127 lipid species including glycerophospholipids, fatty acids, sterols, and sphingolipids. Despite reducing the analyte amount to that of single cells, we identified 15 lipid ions in common shown in Figure S3a and Table S1.

Metabolomic profiling is typically undertaken on many cells using LC-MS, for example. To assess the performance of 3D OrbiSIMS compared to LC-MS data from analysis of many cells, we compared putatively assigned lipids in the LIPID MAPS database from our work using primary macrophages to those detected in the LC-MS study of Abuawad et al. undertaken on a macrophage cell line, which used 6 million cells.^28^ Venn diagram (Figure 2e, Table S1, and Figure S3b) shows how many lipids were obtained in common and how many unique lipid species were detected from LC-MS and 3D OrbiSIMS. For 3D OrbiSIMS data in a negative polarity mode, 47 lipids were identified putatively and 17 of the same lipid compounds were identified in both the 3D OrbiSIMS and LC-MS analysis. In the case of LC-MS studies, 212 putatively annotated lipids were identified in positive and negative polarities including glycerophospholipids, fatty acyls, and fatty acids.^28^ Notably, 30 lipid compounds were putatively identified uniquely using 3D OrbiSIMS, which were not detected in the LC-MS measurement, including fatty acids species FA16:1, FA18:0, FA20:4, and FA22:4 (Table S1).

The identification of some representative putative lipid assignment was confirmed using sequential mass spectrometry analysis in the OrbiTrap (MS/MS). In negative-ion mode spectra, several phosphatidylinositol lipid species were observed in macrophage cells. The product ion spectrum of phosphatidylinositol secondary ions is shown in Figure 2h,i. In the MS/MS spectrum, the precursor ion is [M – H]^−^ at *m*/*z* 861.5494 and [C_45_H_82_O_13_P]^−^ assigned as PI (36:2). The main product ions of the precursor PI (36:2) ion are the signature fragments of the PI head group, [C_6_H_10_PO_8_]^−^ at *m*/*z* 241.0118, and two C18:1 fatty acid moieties are represented by the[C_18_H_33_O_2_]^−^ peak at *m*/*z* 281.3486 (Figure 2h). The structure of PI (38:4) at *m*/*z3* 885.5498 was confirmed based on the detection of the PI head group ions, [C_6_H_10_PO_8_]^−^ at *m*/*z* 241.0, and two fatty acid moieties from these lipids are C18:0, [C_18_H_35_O_2_]^−^ at *m*/*z* 283.2642 and C20:4, [C_20_H_31_O_2_]^−^ at *m*/*z* 303.2327 (Figure 2i). MS/MS spectra of PA and PC lipids are reported in Figure S3c,d and Table S2.

**Figure 3:**
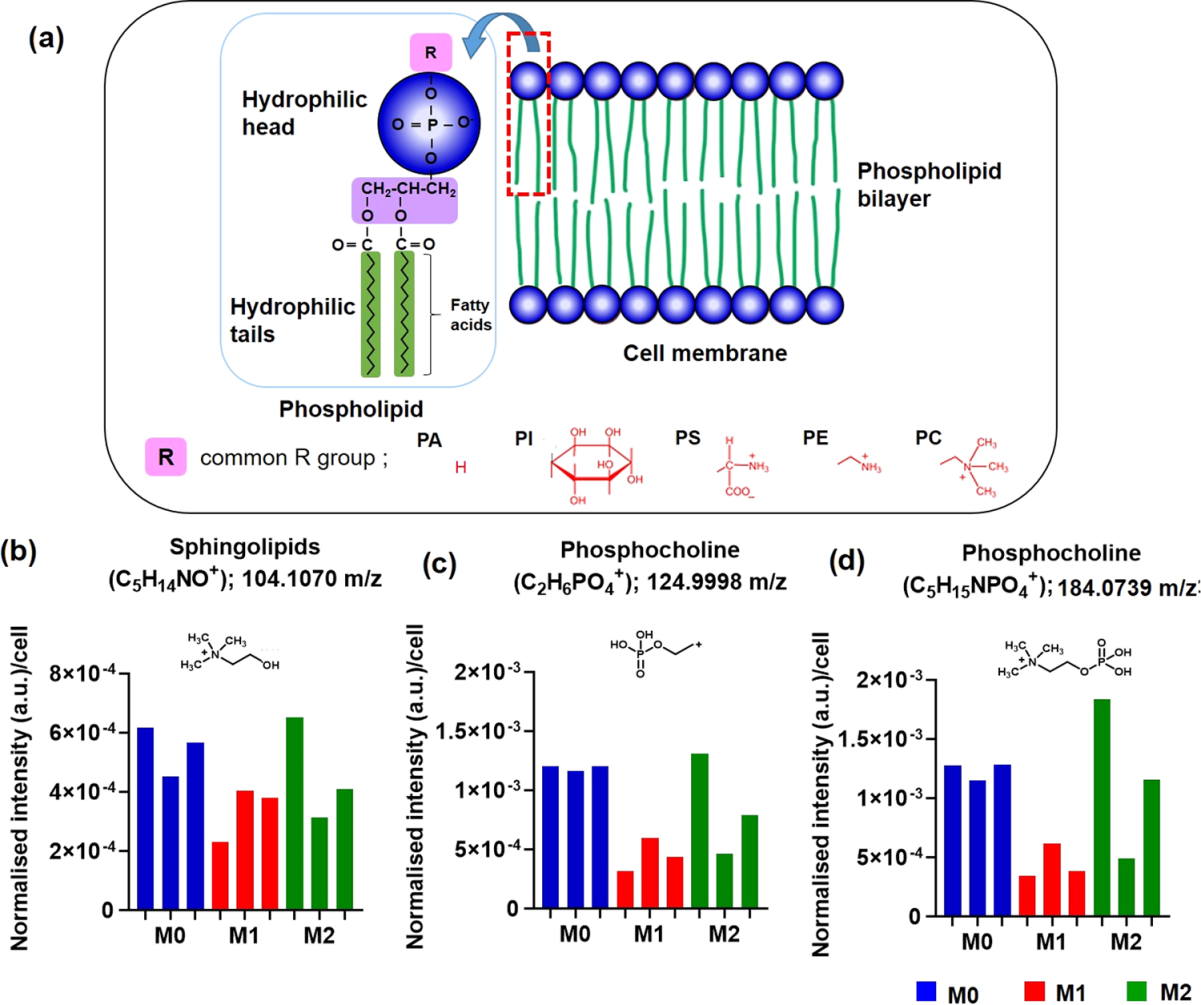
(a) Schematic of the membrane lipid components. Normalized intensity of lipids in three separate cells for each macrophage polarization with comparison of each phenotypes in positive polarity (b) C_5_H_14_NO^+^ (sphingolipids, SP, *m*/*z* 104.1070), (c) C_2_H_6_PO_4_^+^ (phosphocholines, PC, *m*/*z* 124.9998), and (d) C_5_H_15_NPO_4_^+^ (phosphocholines, PC, *m*/*z* 184.0739).

Lipids such as phospholipids and glycolipids are major components of the cell membrane^48^ and key species in understanding metabolic pathways. Phospholipids are the main component of the cell lipid bilayer comprising two long fatty acid chains, a triglyceride linking a phospholipid head with various alkyl groups, namely, ethanolamine, inositol, serine, glycerol, and choline such as phosphatidylethanolamine, phosphatidylinositol, phosphatidylserine, phosphatidylglycerol, and phosphatidylcholine (Figure 3a). Glycerphospholipids have five classes, which are subdivided based on polar head groups (common alkyl groups) including PC, PI, PA, PS, and PE.^48,49^ We found lipids with the head group: PC, PI, PS, and PE, included in Table S3. All phenotypes expressed the lipid head group with a short alkylated chain with ions such as C_5_H_14_NO^+^, C_2_H_6_PO_4_^+^, and C_5_H_15_NPO_4_^+^^50^ (Figure 3b–d and Table S3). Intense negative ions assigned to this class included phosphatidylethanolamine (C_2_H_5_NPO_3_^–^), phosphatidylcholine (C_4_H_11_NPO_4_^–^), phosphatidylglycerol (C_3_H_6_PO_5_^–^), phosphatidylinositol (C_6_H_10_PO_8_^–^), sulfatide (HSO_4_^–^), and sphingolipids (C_2_H_4_PO_4_^–^) (Table S3). Lipid compounds that we found in our samples have previously been observed in the analysis of the individual lipids using TOF-SIMS.^49^

### Untargeted Analysis Approaches

**Figure 4:**
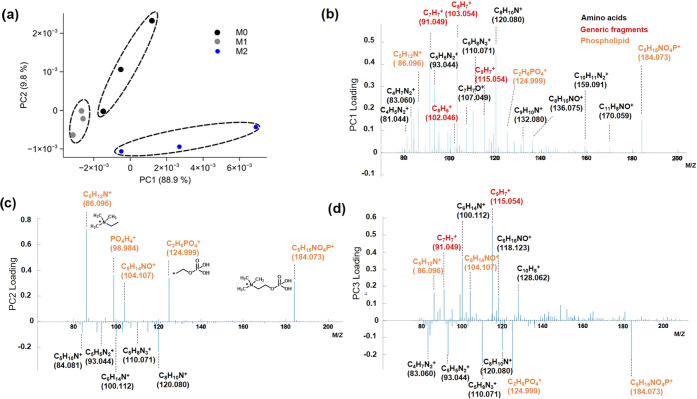
Principal component analysis (scores and loadings) for different macrophage subsets results. (a) Principal component scores plots of PC1 and PC2 for the 3D OrbiSIMS spectra of M0, M1, and M2 macrophage on positive polarity. Loading plot on positive polarity data for the first three principal components; peaks were assigned based on amino acid maker (black), phospholipid maker (orange), and generic, which are nonspecific ion fragments (red). (b) PC1, (c) PC2 and (d) PC3.

To discern subtle differences in the macrophage metabolome and expand beyond the targeted lipid analysis, we undertook an untargeted analysis of the secondary ion data using principal component analysis (PCA) of the three macrophage subsets and their analytical repeats. The resultant scores plot of the first three principal components (PCs) for the positively charged ions in the 3D OrbiSIMS data discriminated all three macrophage subsets and replicate measurements clustered together. Scores (PC1, 2, and 3) and loading plots for three cell types are shown in Figure 4a, and PC4 is shown in Figure S6. Scores revealed molecular similarity between replicates and each macrophage subset shows statistically unique secondary ions, leading to clear chemical separation of each phenotypic macrophage subset. Loadings of the first four components highlighted ions responsible for chemical differences between macrophage types. PC1 showed that amino acids were more intense in the spectra from M2 macrophages (Figure 4a,b). The second component was associated with chemistry from M0 macrophages and included positively ionizing lipid fragments containing CHNOP-containing ions such as C_5_H_15_NO_4_P^+^ (*m*/*z*, 184.07) and C_2_H_6_PO_4_^+^ (*m*/*z*, 124.99) (Figure 4c). The third component differentiated between certain lipids and amino acids (Figure 4d), and scores plots show that this chemistry was most prominent in M1 macrophages (Figures S7 and S8).

### Amino Acid Assignments

**Figure 5:**
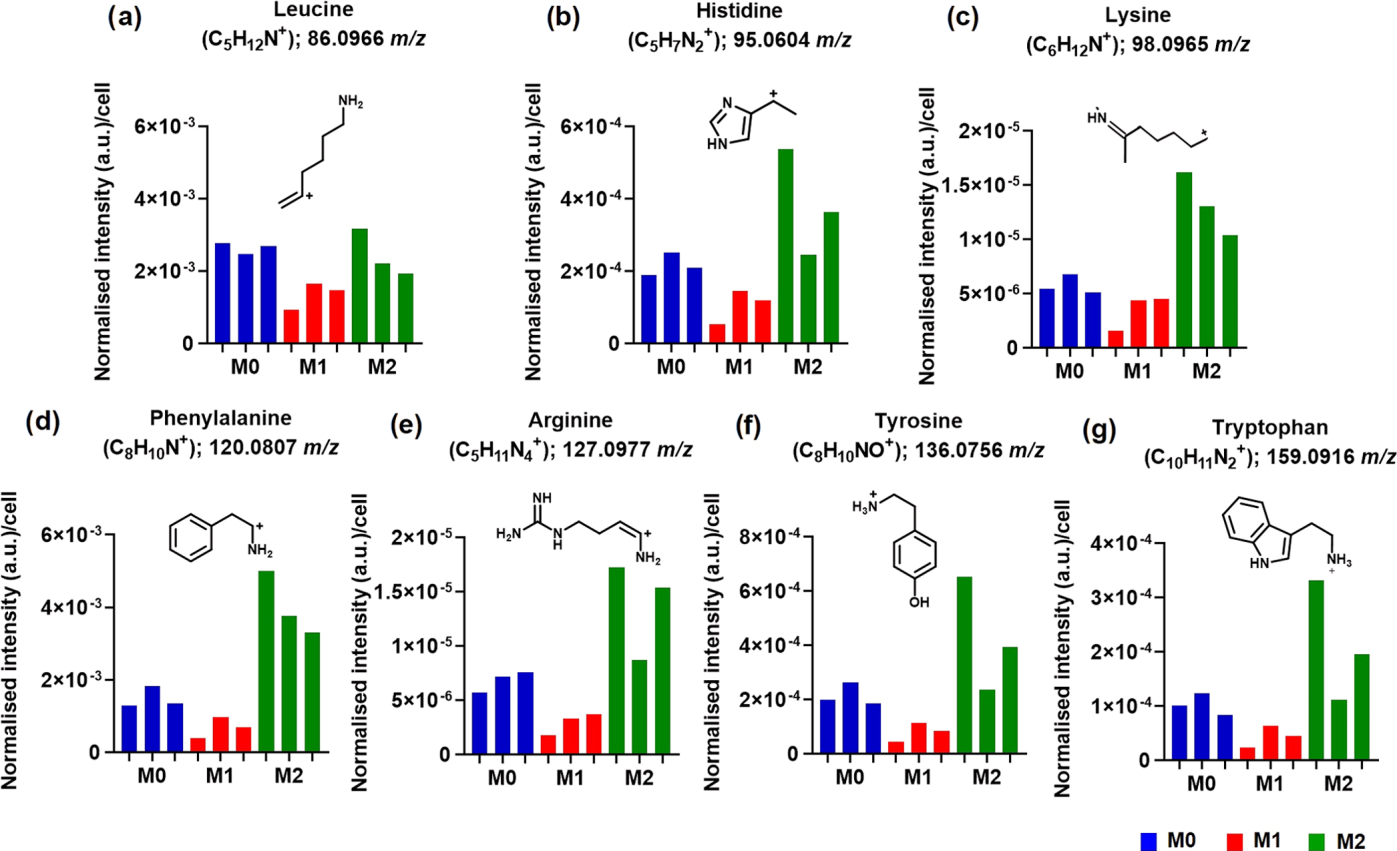
Characteristic amino acid fragments were detected in macrophage polarization in positive-ion mode. The normalized intensity per cell spectral for (a) C_5_H_12_N^+^ (leucine, *m*/*z* 86.0966), (b) C_5_H_7_N_2_^+^ (histidine, *m*/*z* 95.0604), (c) C_6_H_12_N^+^ (lysine, *m*/*z* 98.0965), (d) C_8_H_10_N^+^ (phenylalanine, *m*/*z* 120.0807), (e) C_5_H_11_N_4_^+^ (arginine, *m*/*z* 127.0977), (f) C_8_H_10_NO^+^ (tyrosine, *m*/*z* 136.0756), and (g) C_10_H_11_N_2_^+^ (tryptophan, *m*/*z* 159.0599).

Loadings from PCA were interrogated and identified ions that may be assigned to specific amino acid fragments (Figure 5a–g). Assignments from both polarities were made by comparing fragments identified by TOF-SIMS in previous SIMS studies of amino acids assigned from analysis of proteins and free amino acids.^51−53^ In total, 39 amino acid fragment ions were assigned from the single cells and were present in all samples (Table S4), which we attribute to 13 amino acids from proteins. These include histidine, phenylalanine, which has an important function in immune tolerance controlled by tetrahydrobiopterin synthesis to produce the NO by iNOS in activated macrophages.^54^ Tyrosine is produced by phenylalanine degradation, which is a precursor of melanin synthesis.^55^ Melanin can reduce the pro-inflammatory mediators such as TNFα, IL-1b, IL-6, and IL-10 from monocytes and macrophages, and induce the production of the anti-inflammatory cytokines from leukocytes.^56^ Tryptophan is produced by indoleamine-2,3-dioxygenase (IDO), which converts tryptophan to kynurenine. Tryptophan metabolism could prevent therapeutic targets in treating age-related diseases associated with inflammation and extend health and life span.^57^ Arginine^58^ had the lowest intensity in the M1 macrophage (Figure 5e). M1 macrophages metabolize arginine using the enzyme nitric oxide synthase (NOS) into nitric oxide (NO) and citrulline.^59^ M2 macrophages are known to promote hydrolysis of arginine toward urea and ornithine, which promote proliferation repair. Therefore, amino acid is used differently in M1 and M2 macrophages; it seems to be a key resource to support polarization and function of both M1 and M2 macrophages. This shows that our single-cell metabolomic profiling using 3D OrbiSIMS successfully detected different levels of amino acids and lipids in M0, M1, and M2 and linked the chemical compounds to known macrophage functions. Comparison of ions observed by 3D OrbiSIMS and free amino acids reference spectra acquired by TOF-SIMS did not show matches^51^—suggesting they are derived from larger protein structures (Table S4). In nearly all cases, M2 macrophages showed higher intensity of amino acid fragments than in M0 and M1 (Figure S5 and Table S4).

### Metabolite Identification

**Figure 6:**
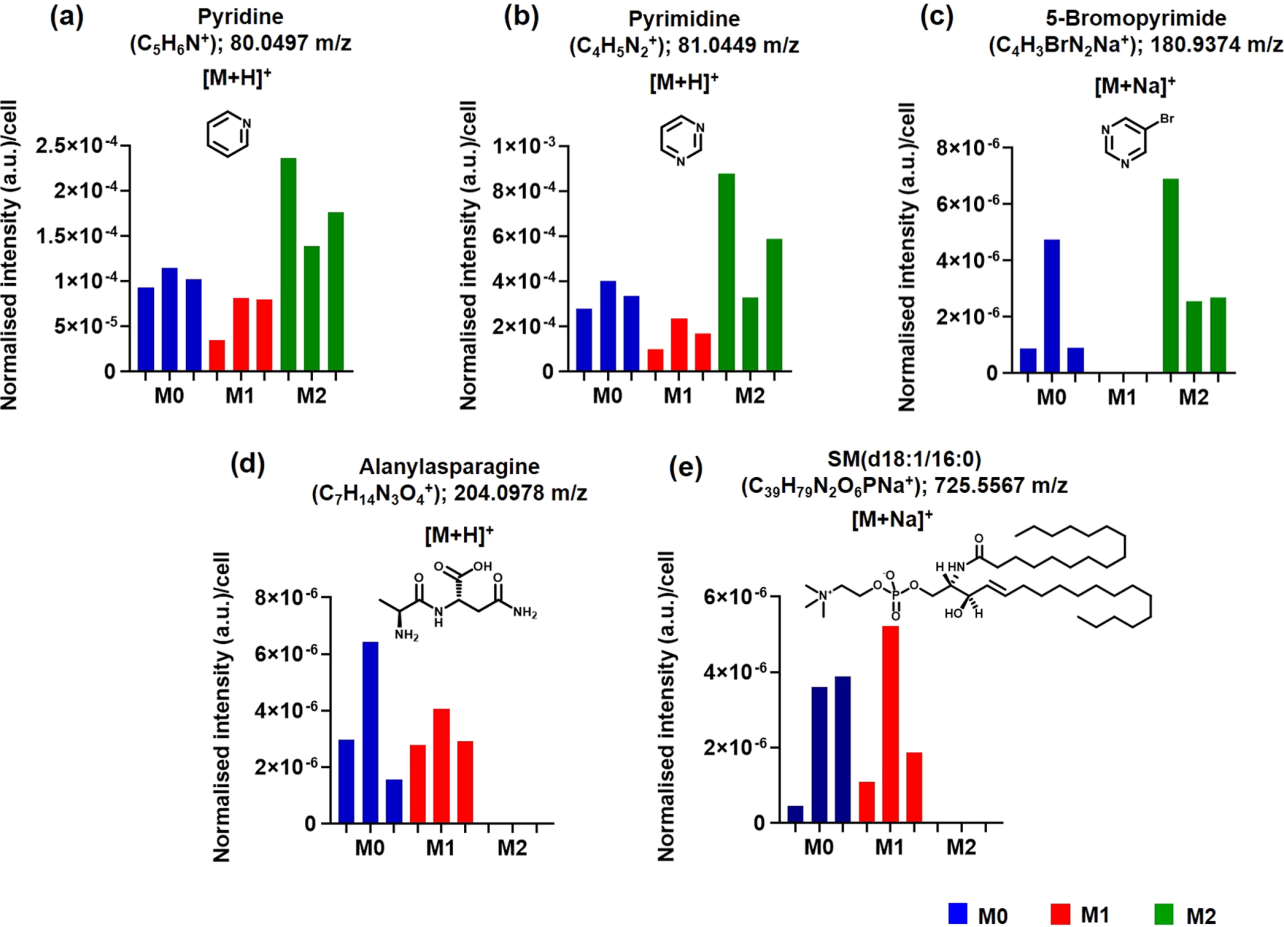
Metabolites significantly affected by macrophage polarization toward M1 and M2. (a) Pyridine [M+H]^+^ at *m*/*z* 80.0497, (b) pyrimidine [M + H]^+^ at *m*/*z* 81.0449, and (c) 5-bromopyridine [M + Na]^+^ at *m*/*z* 180.9374. Pyrimidine metabolisms were observed at increased intensity in M2 macrophages compared to M1 macrophage. (d) Alanylasparagine [M + H]^+^ at *m*/*z* 204.0978, and (e) SM(d18/16:0) [M + Na]^+^ at *m*/*z* 725.5567 are represented uniquely in M1 macrophages.

We correlated ions from 3D OrbiSIMS parent spectra of the single cells to the Human Metabolome Database and found unique metabolites belonging to each subset (Figure 6, Figure S9, and Table S6). For example, 5-bromopyridine [M + Na]^+^ (*m*/*z* 180.9374) had high ion intensity in M2 macrophages but was not observed in the M1 macrophages (Figure 6a–c). The pyridine moiety is an integral part of anti-inflammatory agents; it is known to induce macrophage growth and has been shown to inhibit the formation of tumor necrosis factor stimulants such as lipopolysaccharide.^60^ This is in agreement with our data, which shows the high intensity of pyridine moieties in the anti-inflammatory M2 macrophage compared to no detection in the pro-inflammatory M1 phenotype. This clearly shows how 3D OrbiSIMS data from a single cell could be used to predict macrophage phenotype. The dipeptide alanylasparagine and the lipid sphingomyelin (SM) (d18:1/16:0) were detected uniquely in M1 macrophages. They are both implicated in pro-inflammatory cellular responses^61,62^ and were not found in M2 cells (Figure 6d,e). SM lipids are considered very important for the preservation of immune cell activation and function.^62^ Sphingomyelin is known to covert to ceramide, which plays a key role in inducing pro-inflammatory gene expression with the synergistic effect of LPS.^63^ Moreover, potent M1polarizing cytokines such as TNF-α and IFNγ have been shown to induce ceramide production, which might further augment M1 macrophage polarization.^64^

## Conclusions

In this work, we have shown that metabolites can be detected by direct analysis of single-macrophage cells using a gas cluster primary beam using 3D OrbiSIMS. We found that we could detect differences in the metabolite profiles of naïve (M0), pro-inflammatory (M1), and anti-inflammatory (M2) macrophages. These intensity differences can be linked to the pro- and anti-inflammatory nature of different macrophage types. Coupled with principal component analysis of single-macrophage cells, this analytical technique allowed us to accurately assign several key species including amino acid fragments, lipids, and other small molecules known to play a role in cell metabolism. This approach will allow in situ characterization of single cells to help understand the response of the human body to different environmental insults/stimulations, including exposure to biomaterials, therapeutics, and immune dysregulation from in vitro cell samples and tissue biopsies.

## Supporting Information Available

Concentration of the cytokines for M0, M1, and M2 macrophage activation; lipids and amino acids identified by accurate mass in 3D OrbiSIMS experiment; other supplementary figures and tables (PDF)
Data can be found in the University of Nottingham data repository at https://rdmc.nottingham.ac.uk/handle/internal/9531 (http://doi.org/10.17639/nott.7213)
