# Supplementary material for: Single-Cell Metabolic Profiling of Macrophages Using 3D OrbiSIMS: Correlations with Phenotype
Source: Anal Chem. 2022 Jun 17;94(26):9389–98. doi: 10.1021/acs.analchem.2c01375 (PMC9260720; doi:10.1021/acs.analchem.2c01375)
Supplement: Supplementary file 1 — ac2c01375_si_001.pdf [file ac2c01375_si_001.pdf]

Supporting information

Single cell metabolic profiling of macrophages using 3D OrbiSIMS: correlations with phenotype

Waraporn Suvannapruk,[a] Max K Edney,[b] Dong-Hyun Kim,[a] David J Scurr,[a] Amir M Ghaemmaghami[c] and Morgan R Alexander[a*]

[a] Advanced Materials and Healthcare Technologies Division, School of Pharmacy, University of Nottingham, University Park Nottingham, NG7 2RD, United Kingdom.

[b] Department of Chemical and Environmental Engineering, Faculty of Engineering, University of Nottingham, University Park Nottingham, NG7 2RD, United Kingdom.

[c] Immunology & Immuno-bioengineering Group, School of Life Sciences, Faculty of Medicine and Health Sciences, University of Nottingham, University Park Nottingham, NG7 2RD, United Kingdom.

Corresponding Author: *E-mail: morgan.alexander@nottingham.ac.uk

Contents

Table S1. Putative annotation of lipid compounds in negative ion spectrum of M0, M1 and M2 macrophages.
Table S2. Summary of phospholipids identified in macrophage phenotypes by 3D OrbiSIMS MS/MS in a negative ion mode.

Table S3. Fragments of lipids class in spectra both of positive and negative ions.

Table S4. Molecular ion and fragments of amino acid in macrophage phenotypes from 3D OrbiSIMS spectra.

Table S5. Show unique lipids signature for each phenotype.

Table S6. Metabolite M0, M1 and M2 macrophages search by human metabolome data base.

Figure S1. Macrophage marker expression and cytokines analysis.

Figure S2. 3D OrbiSIMS MS image of the macrophage single cell.

Figure S3. Comparison of the number lipid compounds which detected in single cell macrophages subsets and tissue sample using 3D OrbiSIMS and 3D OrbiSIMS MS/MS spectrum.

Figure S4. Normalised spectra of lipids class in macrophage polarization.

Figure S5. Normalised 3D OrbiSIMS spectrum of amino acids from a single cell macrophage.

Figure S6. PC3 and PC4 scores.

Figure S7. A bar chart showing the proportion of total data variance that is explained by the principal components of negative polarity 3D OrbiSIMS datasets.

Figure S8. Principal component analysis on 3D OrbiSIMS negative polarity datasets.

Figure S9. 3D OrbiSIMS spectrum of metabolite from a single cell macrophage each cell type in positive polarity.

Table S1. Putative annotation of lipid compounds in negative ion spectrum of M0, M1 and M2 macrophages, (* detect in THP- macrophages polarisation by LC-MS methods , ++ detect in tissue section by 3D OrbiSIMS methods, 15 lipid ion in common)

Name	Exact mass (m/z)	Formula $[M-H]^-$	Structure	Information	M0 macrophage		M1 macrophage		M2 macrophage	
					Mass error p.p.m	Area (AU)	Mass error p.p.m	Area (AU)	Mass error p.p.m	Area (AU)
Fatty acids (FA)										
FA 14:0++	227.2029	$C_{14}H_{27}O_2$	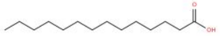	https://www.lipidmaps.org/databases/lmsd/LMFA01010014?LMID=LMFA01010014					-0.3	11520.55
FA 15:0*	241.2180	$C_{15}H_{29}O_2$	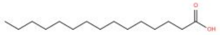	https://www.lipidmaps.org/databases/lmsd/LMFA01010015?LMID=LMFA01010015			0.2	1009	-0.1	10743.61
FA 16:0*,++	255.2331	$C_{16}H_{31}O_2$	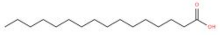	https://www.lipidmaps.org/databases/lmsd/LMFA01010001?LMID=LMFA01010001	0.6	27104.51	0.6	35229.2	0.4	279005.29
FA 16:1++	253.2175	$C_{16}H_{29}O_2$	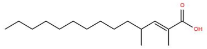	https://www.lipidmaps.org/databases/lmsd/LMFA01020133?LMID=LMFA01020133			0.7	3711.72	0.3	31572.26
FA 17:0*	269.2500	$C_{17}H_{33}O_2$	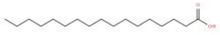	https://www.lipidmaps.org/databases/lmsd/LMFA01010017?LMID=LMFA01010017			1.6	1471.17	0.8	14539.28

FA 17:1	267.2324	$C_{17}H_{31}O_2$	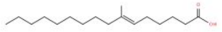	https://www.lipidmaps.org/databases/lmsd/LMFA01020203?LMID=LMFA01020203					0.8	3760.23
FA 18:0++	283.2645	$C_{18}H_{35}O_2$	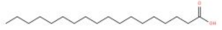	https://www.lipidmaps.org/databases/lmsd/LMFA01010018?LMID=LMFA01010018	0.1	36410	0.1	92587.99	0.1	276538.49
FA 18:1*,++	281.2486	$C_{18}H_{33}O_2$	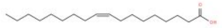	https://www.lipidmaps.org/databases/lmsd/LMFA01030002?LMID=LMFA01030002	-0.1	24011.28	0.0	61453.28	0.1	258961.75
FA 18:2*,++	279.2330	$C_{18}H_{31}O_2$	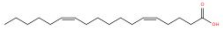	https://www.lipidmaps.org/databases/lmsd/LMFA01030110?LMID=LMFA01030110			-0.2	4568.31	-0.2	22519.37
FA 20:3++	305.2487	$C_{20}H_{33}O_2$	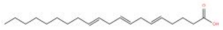	https://www.lipidmaps.org/databases/lmsd/LMFA01030157?LMID=LMFA01030157			0.2	1964.59	0.1	18144.56
FA 20:4++	303.2330	$C_{20}H_{31}O_2$	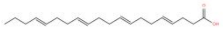	https://www.lipidmaps.org/databases/lmsd/LMFA01030173?LMID=LMFA01030173	0.1	13466.62	0.2	17907.53	-0.2	97974.75
FA 20:5	301.2155	$C_{20}H_{29}O_2$	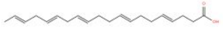	https://www.lipidmaps.org/databases/lmsd/LMFA010301			-1.2	1101.58	-0.7	4707.33

				80?LMID=LMFA01030180						
FA 22:4++	331.2659	$C_{22}H_{35}O_2$	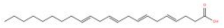	https://www.lipidmaps.org/databases/lmsd/LMFA01030177?LMID=LMFA01030177			-0.4	1367.55		
FA 22:5++	329.2483	$C_{22}H_{33}O_2$	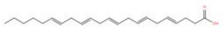	https://www.lipidmaps.org/databases/lmsd/LMFA01030182?LMID=LMFA01030182			-0.1	2114.64	-0.4	13811.97
10,11-Dihydro-12R-hydroxy-leukotriene E4	457.2513	$C_{23}H_{39}NO_6S$	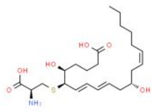	https://www.chemspider.com/Chemical-Structure.30776621.html?rid=38c09e7f-61b7-4979-ab22-a819af4f8fc9&page_num=0	2.0	4412.21	1.8	86282.52	2.0	163681.7
Acyl carnitines (CAR)										
CAR 13:0;O4	420.2605	$C_{20}H_{38}NO_8$	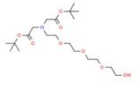	https://www.chemspider.com/Chemical-Structure.29332391.html?rid=d3f9c4dc-e823-49a3-89b1-6764b5d8a075&page_num=0			-0.9	2857.85	-0.3	4585.07
CAR 30:1;O4	656.5096	$C_{37}H_{70}NO_8$	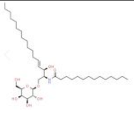	https://www.chemspider.com/Chemical-Structure.9356447.	-1.6	3556.00	-1.0	1675.98		

				html?rid=b7c87349-3c4d-4500-a83d-65fe8798c905						
Phosphatidylinositol (PI)										
PI(18:0/0:0)	599.3216	$C_{27}H_{52}O_{12}P$	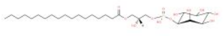	https://www.lipidmaps.org/databases/lmsd/LMGP06050004?LMID=LMGP06050004			0.1	4145.31	0.7	1782.87
PI 18:0-20:3*	887.5661	$C_{47}H_{84}O_{13}P$	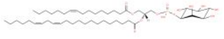	https://www.lipidmaps.org/databases/lmsd/LMGP06010192?LMID=LMGP06010192			0.9	1467.16	0.4	3557.49
PI 34:1*,++	835.5353	$C_{43}H_{80}O_{13}P$	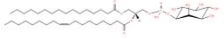	https://www.lipidmaps.org/databases/lmsd/LMGP06010001?LMID=LMGP06010001			0.1	5002.39	-0.4	8495.28
PI 36:1*	863.5556	$C_{45}H_{84}O_{13}P$	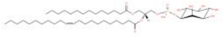	https://www.lipidmaps.org/databases/lmsd/LMGP06010074?LMID=LMGP06010074			-0.4	14522.95	-0.4	18920.7
PI 36:2*	861.5484	$C_{45}H_{82}O_{13}P$	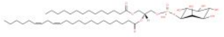	https://www.lipidmaps.org/databases/lmsd/LMGP06010075?LMID=LMGP06010075			-0.5	4305.54	-0.2	10060.63
PI 38:3*,++	883.5316	$C_{47}H_{80}O_{13}P$	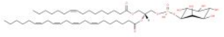	https://www.lipidmaps.org/databases/lmsd/LMGP060101			1.1	1727.55	-0.4	2419.38

				93?LMID=LMGP06010193						
PI 38:4*,++	885.5498	$C_{47}H_{82}O_{13}P$	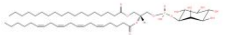	https://www.lipidmaps.org/databases/lmsd/LMGP06010010?LMID=LMGP06010010	0.0	5922.51	-0.3	18814.16	-0.2	61319.66
PI 40:5*	911.5749	$C_{49}H_{84}O_{13}P$	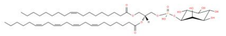	https://www.lipidmaps.org/databases/lmsd/LMGP06010307?LMID=LMGP06010307					0.1	1651.6
Phosphatidylserine (PS)										
PS 36:1*,**	788.5419	$C_{42}H_{79}NO_{10}P$	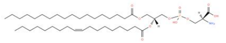	https://www.lipidmaps.org/databases/lmsd/LMGP03010025?LMID=LMGP03010025					-0.4	11220.87
Phosphatidylphosphates (PA)										
PA 18:0	437.2686	$C_{21}H_{42}O_7P$	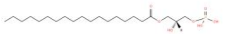	https://www.lipidmaps.org/databases/lmsd/LMGP10050005?LMID=LMGP10050005			-0.9	3121.27	-0.7	9217.13
PA 36:2++	701.5123	$C_{39}H_{74}O_8P$	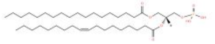	https://www.lipidmaps.org/databases/lmsd/LMGP10010037?LMID=LMGP10010037	-0.6	3262.63	0.0	4862.41	0.0	21753.07
CPA(16:0)*	391.2230	$C_{19}H_{36}O_6P$	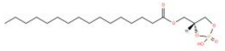	https://www.lipidmaps.org/databases/lmsd/LMGP000000			0.3	1864.02		

				57?LMID=LMGP00000057						
CPA(18:1)	417.2409	$C_{21}H_{38}O_6P$	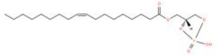	https://www.lipidmaps.org/databases/lmsd/LMGP00000056?LMID=LMGP00000056			-2.0	1259.00	0.1	2988.96
CPA(18:0)	419.2567	$C_{21}H_{40}O_6P$	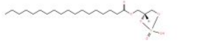	https://www.lipidmaps.org/databases/lmsd/LMGP00000055?LMID=LMGP00000055	-0.3	4362.96	0.0	17874.25	0.1	47590.02
Phosphosphingolipids										
SM(d16:1/17:0)*	687.5443	$C_{38}H_{76}N_2O_6P$	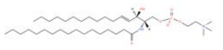	https://www.lipidmaps.org/databases/lmsd/LMSP03010037?LMID=LMSP03010037	-0.5	3554.85	0.1	2446.42		
Sterol lipids										
ST 25:3;O5;G	474.2855	$C_{27}H_{40}NO_6$	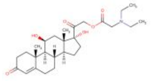	https://hmdb.ca/metabolites/HMDB0014907	-1.4	8322.27	-1.0	200400.34	-0.9	399983.97
ST 24:4;O5;G*	458.2537	$C_{26}H_{36}NO_6$	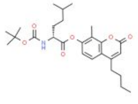	https://www.chemspider.com/Chemical-Structure.60759728.html?rid=05e4d48b-63f7-477a-8156-da70419dd935			-0.5	18364.54	-0.5	33536.62
ST 20:0;O4;Hex	499.2868	$C_{26}H_{43}O_9$	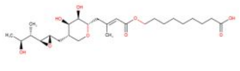	https://hmdb.ca/metabolites/HMDB0014554			1.3	1014.16	1.8	10687.51

ST 22:0;O3;S	443.2328	$C_{22}H_{37}NO_6S$	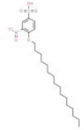	https://www.chemspider.com/Chemical-Structure.3350310.html?rid=130191b8-3c36-46d9-b3d4-296830171ba3&page_num=0			1.4	1886.76		
ST 22:1;O3;S	441.2198	$C_{22}H_{35}NO_6S$	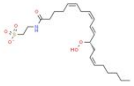	https://www.chemspider.com/Chemical-Structure.58170237.html?rid=037a593e-79b6-4af1-a59c-6390522403f6&page_num=0			1.0	2408.43		
ST 24:0;O3;S	471.2636	$C_{24}H_{41}NO_6S$	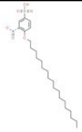	https://www.chemspider.com/Chemical-Structure.4409329.html?rid=bfff8ff0-bfb2-4dce-a96c-3e3ae452f9b4&page_num=0			0.5	1474.87		
ST 21:0;O6;T	490.2467	$C_{23}H_{40}NO_8S$	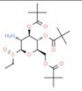	https://www.chemspider.com/Chemical-Structure.9180690.html?rid=094670ed-8195-4a32-b287-aa54719e317d			0.9	2238.07		
ST 24:1;O4;T*	498.2899	$C_{26}H_{44}NO_6S$	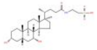	https://www.lipidmaps.org/databases/lmsd/LMST050400			0.8	1886.64	0.1	17797.04

				05?LMID=LMST05040005						
Oxidized glycerophosphoglycerols										
POV-PG	581.3093	$C_{27}H_{50}O_{11}P$	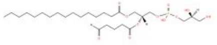	https://www.lipidmaps.org/databases/lmsd/LMGP20060015?LMID=LMGP20060015			-0.3	10462.39	-0.4	17074.60
Neutral glycosphingolipids										
HexCer 31:0;O3	674.5200	$C_{37}H_{72}NO_9$	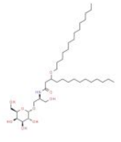	https://www.chemspider.com/Chemical-Structure.8592531.html?rid=741950d8-7717-4e0c-8da7-d5ad707948b4&page_num=0	-1.8	5835.93	-1.2	3370.13	-1.7	6039.87
HexCer 32:1;O4	702.5152	$C_{38}H_{72}NO_{10}$	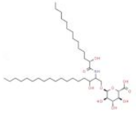	https://www.chemspider.com/Chemical-Structure.8093797.html?rid=1d47af1c-9779-475d-b311-e2257580b65b&page_num=0			0.3	1225.62		
Hex2Cer 33:6;O2	836.5296	$C_{45}H_{75}NO_{13}$	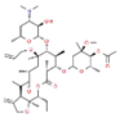	http://www.chemspider.com/Chemical-Structure.8661076.html?rid=efc8a648-46ce-45c7-81ef-93db23a492c3&page_num=0			-0.4	1629.11	-1.1	2151.49

HexCer 42:6;O6	864.5605	$C_{44}H_{82}NO_{12}$	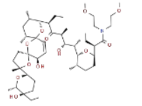	http://www.chemspider.com/Chemical-Structure.58989989.html?rid=76203e10-d373-481f-a6c1-ea3ac92770e5			-0.3	8612.37	-0.4	8940.41
Hex2Cer 31:3;O5	862.5459	$C_{43}H_{76}NO_{16}$	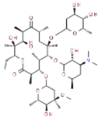	http://www.chemspider.com/Chemical-Structure.34524087.html?rid=5e63d254-76da-42fd-bad5-2925cb83a702			-0.5	2467.17	-0.7	2598.76
Hex2Cer 36:2;O2	886.5529	$C_{48}H_{88}NO_{13}$	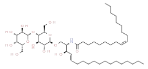	http://www.chemspider.com/Chemical-Structure.24765751.html?rid=de2bc5ec-b095-4640-b2c8-ea5bc80867cd&page_num=0			0.1	10416.22	-0.3	28614.61

Table S2. Summary of phospholipids identified in macrophage phenotype by 3D OrbiSIMS MS/MS in a negative ion mode**.**

**Phosphatidylinositol PI 38:4 (P-18:0/20:4)**		
m/z	Formula	ID
241.0118	$C_6H_{10}O_8P^-$	Head group
283.2642	$C_{18}H_{35}O_2^-$	FA[18:0-H]$^-$
303.2327	$C_{20}H_{31}O_2^-$	FA[20;4-H]$^-$
885.5498	$C_{47}H_{82}O_{13}P^-$	M-H$^-$

**Phosphatidylinositol PI 36:2 (18:0/18:2)**		
m/z	Formula	ID
241.0118	$C_6H_{10}O_8P^-$	Head group
281.2486	$C_{18}H_{33}O_2^-$	FA[18:1-H]$^-$
861.5494	$C_{45}H_{82}O_{13}P^-$	M-H$^-$

**Phosphatidylphosphate CPA 18:0**		
m/z	Formula	ID
152.9950	$C_3H_6O_5P^-$	Head group
283.2642	$C_{18}H_{35}O_2^-$	FA[18:0-H]$^-$
419.2567	$C_{21}H_{40}O_6P^-$	M-H$^-$

**Sphingomyelin SM(d16:1/17:0)**		
m/z	Formula	ID
168.0430	$C_4H_{11}NO_4P^-$	Head group
269.2486	$C_{17}H_{33}O_2$-	FA[17:0-H]$^-$
687.544	$C_{38}H_{76}NO_4P^-$	M-H$^-$

Table S3. Characteristic fragments of lipids class in spectra both of positive and negative ions.

Exact mass (m/z)	Assignment	Amino acids	M0 macrophage		M1 macrophage		M2 macrophage	
			Mass error p.p.m	Intensity (AU)	Mass error p.p.m	Intensity (AU)	Mass error p.p.m	Intensity (AU)
**PA**								
122.0007	$C_2H_5NPO_3^-$	Phosphoethanolamine	0.0	13284.48	-0.3	10267.38	0.4	16141.54
**PC**								
124.9998	$C_2H_6PO_4^+$	Phosphocholines	-0.4	1967962.06	-0.1	383873.28	-0.5	824104.54
184.0739	$C_5H_{13}NPO_4^+$	Phosphocholines	-0.5	1857923.96	-0.4	383873.23	-0.8	588869.19
168.0429	$C_4H_{11}NPO_4^-$	Phosphocholines	-1.1	3488.00	1.2	3643.00	-0.8	17622.00
**PG**								
152.9957	$C_3H_6PO_5^-$	Phosphatidylglycerols	-0.9	6709.45	-0.7	18474.48	-0.8	46522.86
**PI**								
241.0119	$C_6H_{10}PO_8^-$	Phosphatidylinositol	0.3	6709.00	0.2	18518.00	0.3	46586.00
**Sulfatide**								
96.9660	HSO_4^-	Sulfatide	-0.9	950273.38	-1.1	249561.18	-1.1	291906.45
**Sphingolipids**								
104.1070	$C_5H_{14}NO^+$	Sphingolipids	-0.1	955132.00	-0.7	53874.00	-0.6	29350.00
122.9830	$C_2H_4PO_4^-$	Sphingolipids	2.3	227.00	-0.3	18497.00	-0.4	49393.00

Table S4. Characteristic molecular ion and fragments of amino acid in 3D OrbiSIMS spectra.

Exact mass (m/z)	Assignment	Amino acids	M0 macrophage		M1 macrophage		M2 macrophage	
			Mass error p.p.m	Intensity (AU)	Mass error p.p.m	Intensity (AU)	Mass error p.p.m	Intensity (AU)
Positive								
86.0966	$C_5H_{12}N^+$	Leucine	2.0	4268839.21	2.0	1029255.17	1.6	2445367.60
81.0449	$C_4H_5N_2^+$	Histidine	2.4	428865.21	2.4	146241.30	2.0	392938.68
82.0527	$C_4H_6N_2^+$	Histidine	1.9	81132.91	2.0	23550.63	1.8	48841.26
83.0606	$C_4H_7N_2^+$	Histidine	2.2	905324.65	2.3	275942.28	1.9	784924.33
93.0448	$C_5H_5N_2^+$	Histidine	1.2	1677517.08	1.3	563937.95	0.9	1561973
94.0526	$C_5H_6N_2^+$	Histidine	0.7	9340.83	0.5	1942.23	1.0	6855.31
95.0604	$C_5H_7N_2^+$	Histidine	0.7	293808.21	0.8	91140.71	0.5	240892.58
84.0446	$C_4H_6NO^+$	Glutamic acids	2.0	360845.09	2.2	80797.67	1.8	303815.74
84.0810	$C_5H_{10}N^+$	Lysine	2.1	683779.2	2.1	187785.78	1.8	754508.03
96.0808	$C_6H_{10}N^+$	Lysine	0.5	35752.77	0.6	12291.68	0.4	31007.58
98.0965	$C_6H_{12}N^+$	Lysine	1.1	8394.46	0.1	2693.80	-0.3	7228.64
86.0601	$C_4H_8NO^+$	Hydroxyproline	1.6	5974.06	2.6	954.91	1.0	4416.48
118.0650	$C_8H_7O^+$	Phenylalanine	-0.2	504576.41	-0.3	151938.27	-0.7	458877.85
120.0807	$C_8H_{10}N^+$	Phenylalanine	-0.5	1989542.7	-0.5	599777.58	-0.9	2235680.19
121.0647	$C_8H_9O^+$	Phenylalanine	-0.7	8243.94	-0.4	2368.80	-0.9	7618.23
98.0237	$C_4H_4NO_2^+$	Asparagine	1.5	6988.88	0.0	1600.15	0.1	5574.41
100.0869	$C_4H_{10}N_3^+$	Arginine	0.3	8686.98	0.6	2413.67	0.1	9960.78
110.0712	$C_5H_8N_3^+$	Arginine	-0.5	1014232.03	-0.5	264559.98	-0.9	943230.8
112.0868	$C_5H_{10}N_3^+$	Arginine	-0.9	28856.67	-0.9	8970.61	-1.3	17015.06
127.0978	$C_5H_{11}N_4^+$	Arginine	-0.9	8731.00	-0.4	2034.00	-0.9	7716.00
107.0491	$C_7H_7O^+$	Threonine	-0.4	530421.32	-0.4	187460.56	-0.9	564306.94
117.0572	$C_8H_7N^+$	Tryptophan	-0.8	475079.27	-0.8	174457.23	-1.2	370176.13
120.0444	$C_7H_6NO^+$	Tryptophan	-0.3	31388.02	-0.4	13576.44	-0.7	31572.26
130.0651	$C_9H_8N^+$	Tryptophan	-0.3	2692601.54	-0.4	2692601.54	-0.7	2794719.52
132.0806	$C_9H_{10}N^+$	Tryptophan	-0.6	396541.56	-0.6	396541.56	-1.1	337720.8
157.0759	$C_{10}H_9N_2^+$	Tryptophan	-0.7	31002.22	-0.6	11047.56	-1.0	20794.57
158.0836	$C_{10}H_{10}N_2^+$	Tryptophan	-1.0	15252.33	-0.3	4563.37	-1.1	12266.31
159.0916	$C_{10}H_{11}N_2^+$	Tryptophan	-0.7	156283.59	-0.7	39548.78	-1.1	148183.53
170.0599	$C_{11}H_8NO^+$	Tryptophan	-0.7	147148.99	-0.8	37508.96	-1.2	142721.79
131.0491	$C_9H_7O^+$	Tyrosine	-0.8	14207.59	-0.6	6063.81	-1.1	10037.3
136.0757	$C_8H_{10}NO^+$	Tyrosine	-0.3	307949.99	-0.3	71204.87	-0.7	292039.96
143.0729	$C_{10}H_9N^+$	Tyrosine	-0.5	156282.11	-0.5	65032.31	-0.9	143203.51
80.0497	$C_5H_6N^+$	Leucine	2.4	143515.88	2.4	50761.58	1.8	105975.73
89.0387	$C_7H_5^+$	Generic fragment	1.6	285219.17	1.6	128542.2	1.2	203516.01
91.0499	$C_7H_7^+$	Generic fragment	1.4	2867598.83	1.5	1089582.69	1.1	2445367.60
102.0464	$C_8H_6^+$	Generic fragment	0.2	257110.43	0.2	109913.98	-0.3	176507.47

103.0542	$C_8H_7^+$	Generic fragment	0.0	3401198.8	0.0	1289107.03	-0.4	2824757.5
105.0698	$C_8H_9^+$	Generic fragment	-0.3	143062.27	-0.4	54402.24	-0.7	92956.82
117.0572	$C_8H_8N^+$	Generic fragment	-0.9	291934.15	-0.9	107415.65	-1.3	252342.79
Negative								
81.0455	$C_4H_5N_2^-$	Generic	-3.7	13447.05	-4.3	6158.27	-4.5	5764.27
82.0295	$C_4H_4NO^-$	Multiple amino acids	-3.9	42953.75	-4.1	24967.88	-4	26289.81
87.0086	$C_3H_3O_3^-$	Generic	-2.5	19349.63	-3	13630.85	-2.9	12496.62
93.0344	$C_6H_5O^-$	Multiple amino acids	-1.6	221874.12	-1.6	143277.52	-1.6	172089.69
93.0457	$C_5H_5N_2^-$	Multiple amino acids	-1.5	152901.43	-1.5	64905.94	-1.8	86316.64
97.0083	C_8H^-	Generic	-0.9	139922.84	-1	102640.84	-1.1	128993
98.0247	$C_4H_4NO_2^-$	Multiple amino acids	-0.8	156072.36	-0.6	30090.86	-1	39461.92
99.0087	$C_4H_3O_3^-$	Generic	-0.7	9056.85	-1.2	3475.35	-0.6	2379.78
99.0563	$C_4H_7N_2O^-$	Multiple amino acids	-0.4	15224.83	-0.6	6868.53	-0.4	9753.44
100.0404	$C_4H_6NO_2^-$	Multiple amino acids	-0.4	10370.05	-0.5	4504.56	-0.6	4189.82
108.0455	$C_6H_6NO^-$	Multiple amino acids	0.2	27212.24	0	19465.43	-0.1	20992.92
108.0567	$C_5H_6N_3^-$	Histidine	0.1	31897.4	0.1	11492.48	0	9320.24
110.0248	$C_5H_4NO_2^-$	Multiple amino acids	0	80874.52	-0.3	39262.47	-0.2	48553.79
112.0404	$C_5H_6NO_2^-$	Multiple amino acids	0	50993.13	0	20200.69	-0.1	17039.8
113.0357	$C_4H_5N_2O_2^-$	Generic	0.2	69892.94	0	29246.45	0.1	40773.6
116.0506	$C_8H_6N^-$	Multiple amino acids	-0.2	450076.59	-0.3	298477.35	-0.3	365321.71
118.0662	$C_8H_8N^-$	Multiple amino acids	0	59255.9	-0.3	41356.12	-0.3	49696.81
119.0502	$C_8H_7O^-$	Generic	-0.6	1244476.29	-0.6	549492.19	-0.7	621331
128.0353	$C_5H_6NO_3^-$	Multiple amino acids	-0.4	56823.82	-0.4	5812.54	-0.9	21709.08
134.0611	$C_8H_8NO^-$	Generic	-0.6	85468.4	-0.6	95478.63	-0.7	96558.98

Table S5. Show unique lipids signature for each phenotype; 7 unique for M1 and 4 unique for M2.

Mass m/z	Name	Formula $[M-H]^-$	Structure	Information	Mass error p.p.m	Area (AU)
M1						
331.2659	Fatty acids FA (22:4)<br>Adrenic acid	$C_{22}H_{35}O_2$	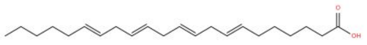	https://www.lipidmaps.org/databases/lmsd/LMFA04000050?LMID=LMFA04000050	-0.4	1367.55
391.2230	CPA(16:0)<br>1-hexadecanoyl-sn-glycero-2,3-cyclic-phosphate	$C_{19}H_{36}O_6P$	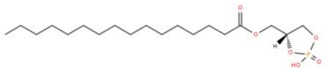	https://www.lipidmaps.org/databases/lmsd/LMGP00000057?LMID=LMGP00000057	0.3	1864.02
441.2198	ST 22:1;O3;S<br>2-{[(5Z,8Z,10E,12S,14Z)-12-Hydroperoxy-5,8,10,14 icosatetraenoyl]amino}ethanesulfonate	$C_{22}H_{35}NO_6S$	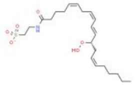	https://www.chemspider.com/Chemical-Structure.58170237.html?rid=037a593e-79b6-4af1-a59c-6390522403f6&page_num=0	1.0	2408.43
443.2328	ST 22:0;O3;S<br>4-(Hexadecyloxy)-3-nitrobenzenesulfonic acid	$C_{22}H_{37}NO_6S$	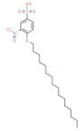	https://www.chemspider.com/Chemical-Structure.3350310.html?rid=130191b8-3c36-46d9-b3d4-	1.4	1886.76

				296830171ba3&page_num=0		
471.2636	ST 24:0;O3;S<br>3-Nitro-4 (octadecyloxy)benzenesulfonic acid	$C_{24}H_{41}NO_6S$	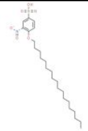	https://www.chemspider.com/Chemical-Structure.4409329.html?rid=bfff8ff0-bfb2-4dce-a96c-3e3ae452f9b4&page_num=0	0.5	1474.87
490.2467	ST 21:0;O6;T<br>(1S)-2-Amino-1,5-anhydro-2-deoxy-3,4,6-tris-O-(2,2-dimethylpropanoyl)-1-[(S)-ethylsulfinyl]-D-glucitol	$C_{23}H_{40}NO_8S$	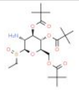	https://www.chemspider.com/Chemical-Structure.9180690.html?rid=094670ed-8195-4a32-b287-aa54719e317d	0.9	2238.07
702.5152	HexCer 32:1;O4<br>Tetradecanamide	$C_{38}H_{72}NO_{10}$	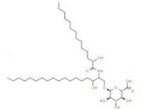	https://www.chemspider.com/Chemical-Structure.8093797.html?rid=1d47af1c-9779-475d-b311-e2257580b65b&page_num=0	0.3	1225.62
M2						
227.2029	Fatty acid FA (14:0) Tetradecanoic acid	$C_{14}H_{27}O_2$	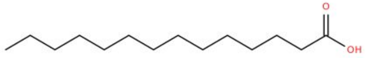	https://www.lipidmaps.org/databases/lmsd/LMFA01	-0.3	11520.55

				010014?LMID=L MFA01010014		
267.2324	Fatty acid FA (17:1) 2-heptadecylenic acid	$C_{17}H_{31}O_2$	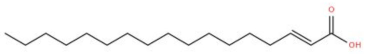	https://www.lipidmaps.org/databases/lmsd/LMFA01030059?LMID=LMFA01030059	0.8	3760.23
788.5419	PS (36:1) 1-octadecanoyl-2-(9Z-octadecenoyl)-sn-glycero-3-phosphoserine	$C_{42}H_{79}NO_{13}P$	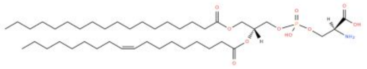	https://www.lipidmaps.org/databases/lmsd/LMGP03010025?LMID=LMGP03010025	-0.4	11220.87
911.5749	PI (40:5) 1-(9Z-octadecenoyl)-2-(7Z,10Z,13Z,16Z-docosatetraenoyl)-glycero-3-phospho-(1'-myo-inositol)	$C_{49}H_{84}O_{13}P$	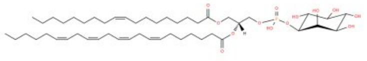	https://www.lipidmaps.org/databases/lmsd/LMGP06010307?LMID=LMGP06010307	0.1	1651.6

Table S6. Metabolite M0, M1 and M2 macrophages search by human metabolome data base

Name	Mass m/z	Adduct	Structure	Human metabolome data base (ID)	M0 macrophage		M1 macrophage		M2 macrophage	
					Mass error p.p.m	Area (AU)	Mass error p.p.m	Area (AU)	Mass error p.p.m	Area (AU)
Pyridine	80.0496	[M+H]	C_5H_6N	HMDB0000926	2.4	144905.32	2.4	51158.97	1.8	106529.37
Pyrimidine	81.0449	[M+H]	$C_4H_5N_2$	HMDB0003361	2.4	431385.01	2.4	147450.11	2.0	396498.09
N-Nitroso-pyrrolidine	83.0605	[M+H-H2O]	$C_4H_7N_2$	HMDB0031642	2.2	907701.19	2.3	276877.39	1.9	787296.88
1Aminocyclopropanec arboxylic acid	84.0445	[M+H-H2O]	C_4H_6NO	HMDB0036458	2.0	363612.61	2.2	81270.59	1.8	306227.15
Piperidine	86.0965	[M+H]	$C_5H_{12}N$	HMDB0034301	2.0	4294975.47	2.0	1035148.99	1.6	1425026.04
Methcathinone	91.0543	[M+H+NH$_4$]	$C_{10}H_{13}NO$	HMDB0041927	1.4	2888641.07	1.5	1099076.46	1.1	2469613
5-Bromopyrimidine	180.9374	[M+Na]	$C_4H_3N_2NaBr$	HMDB0062280	1.3	1340.51	3.6	0	0.9	3084.15
Alanylasparagine	204.0977	[M+H]	$C_7H_{14}N_3O_4$	HMDB0028682	-0.7	6796.17	-0.3	2872.9	6.0	0
SM(d18:1/16:0)	725.5558	[M+Na]	$C_{39}H_{79}N_2O_6PNa$	HMDB0010169	-1.3	3805.42	-1.0	1118.04	5.7	0

a)

Cell types	The number of cells	Concentration of cytokines
M0	1×10^5	20ng/ml M-CSF
M1	1×10^5	50ng/ml GM-CSF+20ng/ml IFNγ
M2	1×10^5	50ng/ml M-CSF+ 20ng/ml IL-4

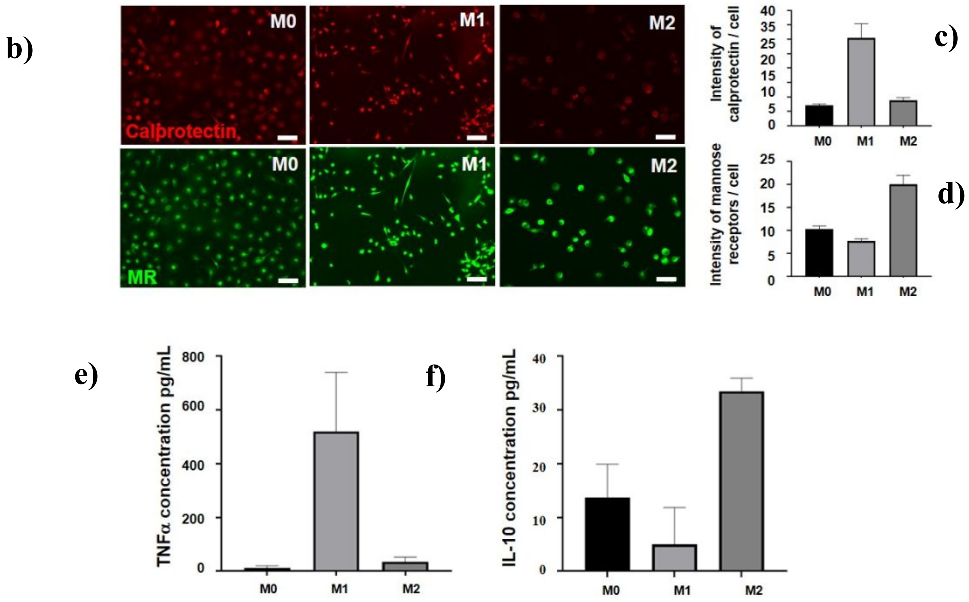

Figure S1. a) The concentration of the cytokines for M0, M1 and M2 macrophages activation. Fluorescent images of macrophages stained for calprotectin and mannose receptor. b) Calprotectin (red), and mannose receptor (green), images were taken under 20 x magnification and scale bar = 50 µm. c) The intensity of calprotectin per cell in macrophage images. d) The intensity of mannose receptor per cell in macrophage images. Comparison of cytokines production by different macrophage subsets, supernatants of each macrophage subsets cultured for 6 days were measured by the enzyme-linked immunosorbent assay. e) TNF-α and f) IL-10; N=3. The intensity of cell on each slide was quantified using CellProfiler.

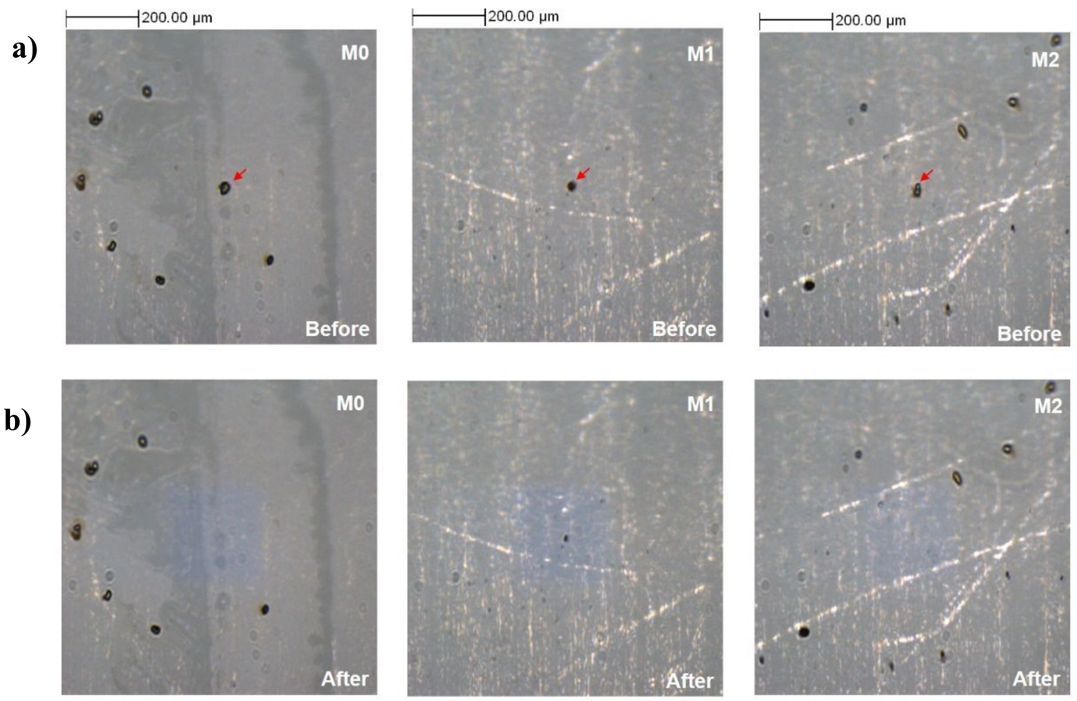

Figure S2. 3D OrbiSIMS MS image of the macrophage single cell a) before and b) after analysis. Images were taken over the area 500 x 500 µm and scale bar = 200 µm.

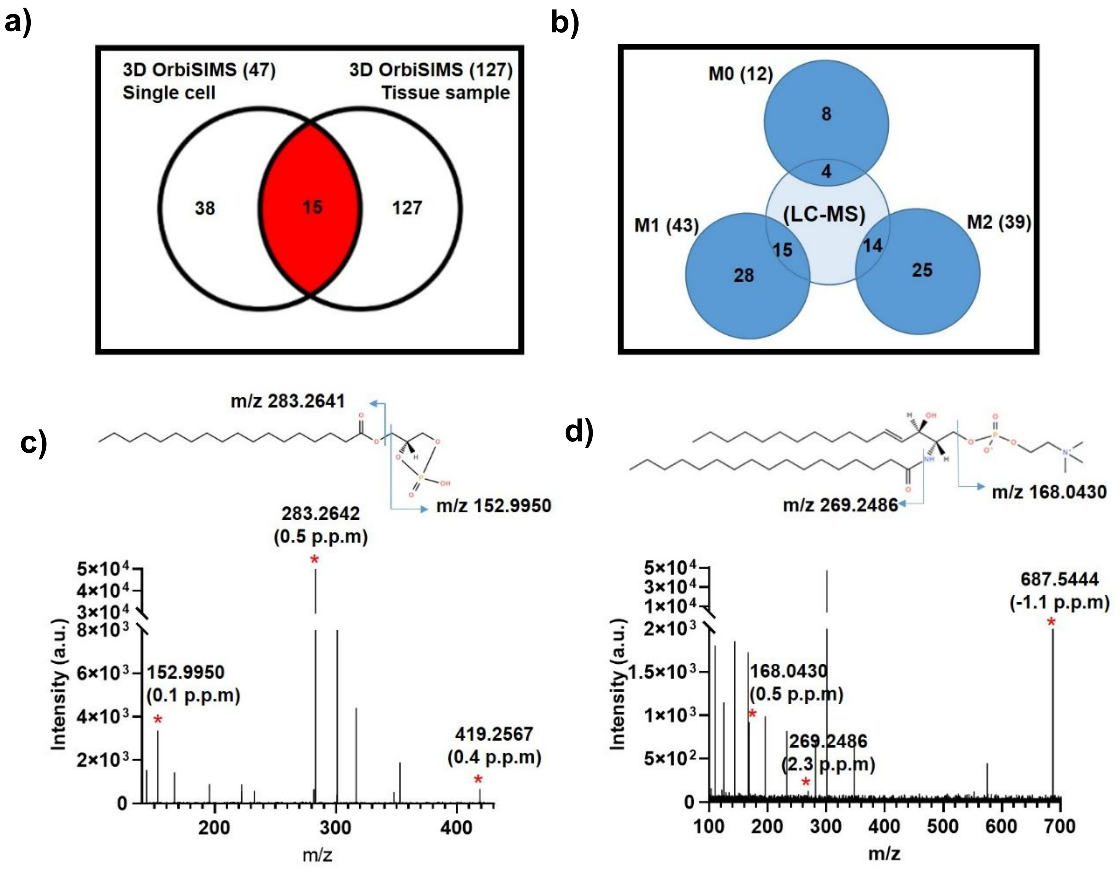

Figure S3. a) Venn diagram comparison of the number lipid compounds which detected in single cell macrophages subsets and tissue sample using 3D OrbiSIMS. b) Comparison the number of lipids compounds in M0, M1 and M2 macrophages using 3D OrbiSIMS and macrophages using LC-MS[1]. Abuawad et al identified, 212 lipids in THP-1 macrophages cell line extracts using LC-MS. The number of lipids were identified in macrophages polarisation using 3D OrbiSIMS, the lipids was mainly number of M0 macrophage (12), M1 macrophage (43) and M2 macrophage (39). Seventeen of lipids compound was the same species in 3D OrbiSIMS and LC-MS protocol. Negative ion mode 3D OrbiSIMS MS/MS spectrum of the precursor ion [M-H]- at m/z 419.2567, $[C_{21}H_{40}O_6P]^-$ recognized as CPA (18:0) c) and SM at m/z 687.5444, $[C_{38}H_{76}NO_4P]^-$ d)

[1] Abuawad, A.; Mbadugha, C.; Ghaemmaghami, A. M.; Kim, D.-H., Metabolic characterisation of THP-1 macrophage polarisation using LC–MS-based metabolite profiling. *Metabolomics* **2020,** *16* (3), 33

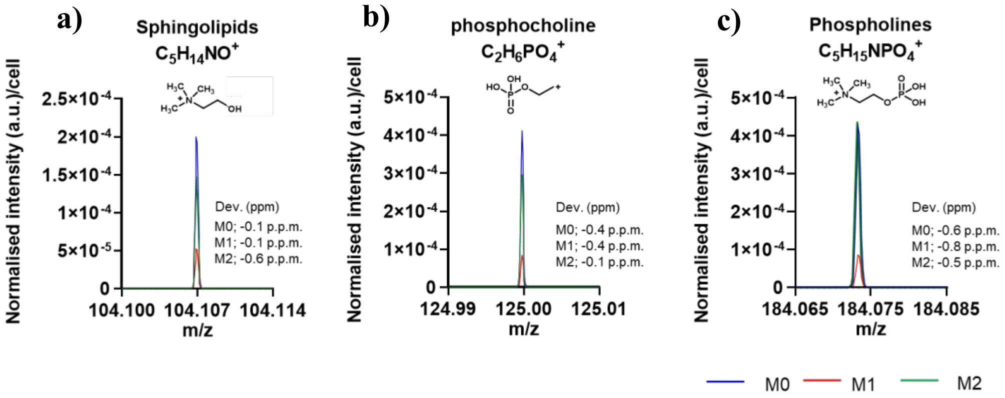

Figure S4. 3D OrbiSIMS of lipid class from a single cell macrophage. Normalised spectra of lipids in macrophage polarisation with comparison of each phenotypes in positive polarity. a) $C_5H_{14}NO^+$ (sphingolipids, SP, m/z 104.1070), b) $C_2H_6PO_4^+$ (phosphocholines, PC, m/z 124.9998) and c) $C_5H_{15}NPO_4^+$ (phosphocholines, PC, m/z 184.0739).

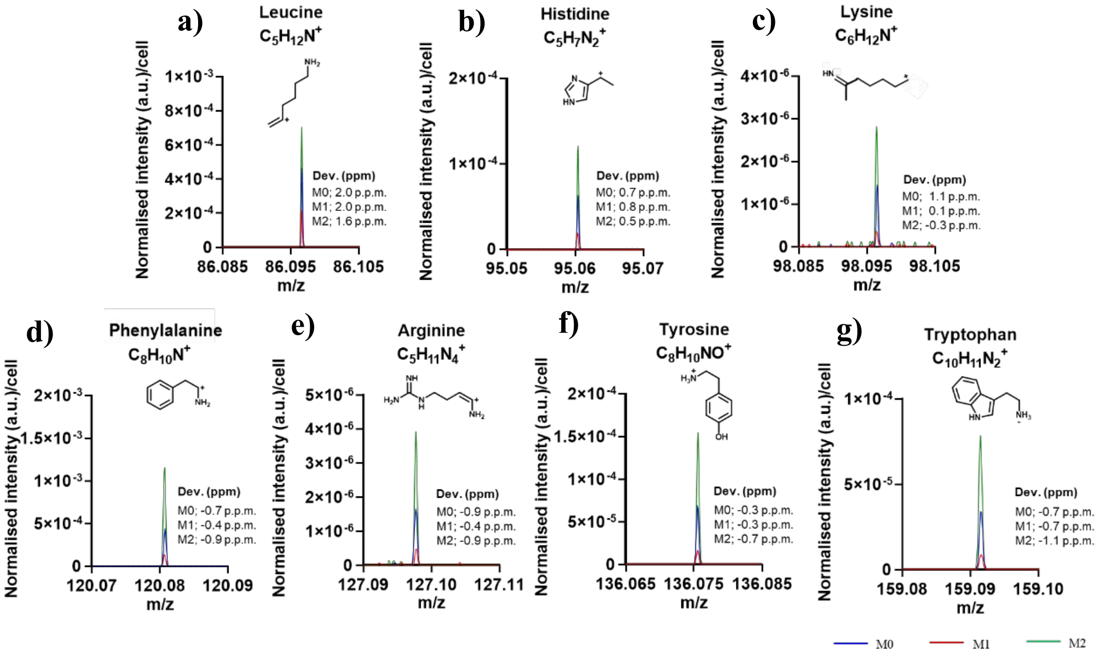

Figure S5. 3D OrbiSIMS spectrum of amino acids from a single cell macrophage. Normalised spectral of amino acids in macrophage polarisation with comparison of each phenotypes in positive polarity a) leucine ($C_5H_{12}N^+$), b) histidine ($C_5H_7N_2^+$), c) lysine ($C_6H_{12}N^+$), d) phenylalanine ($C_8H_{10}N^+$), e) arginine ($C_5H_{11}N_4^+$), f) tyrosine ($C_8H1_0NO^+$), g) tryptophan ($C_{10}H_{11}N_2^+$).

Positive polarity

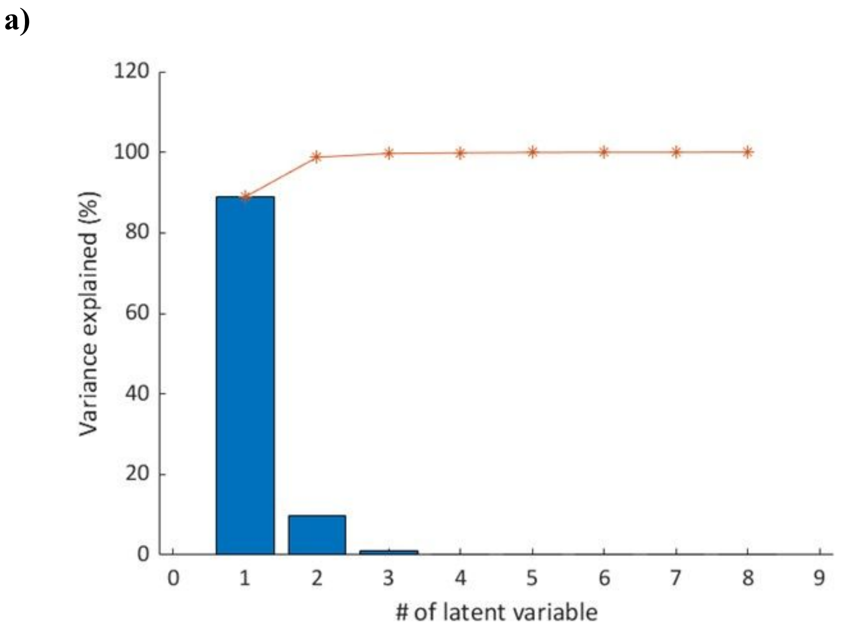

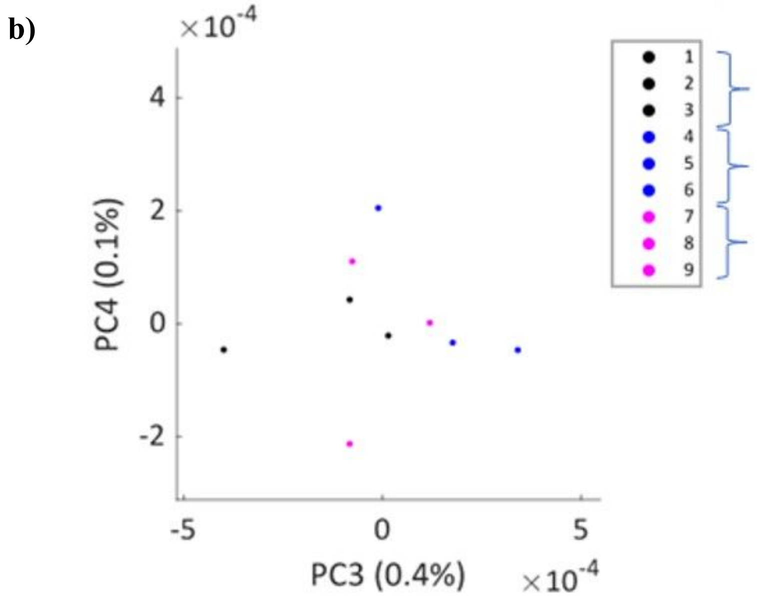

Figure S6. a) A bar chart showing the proportion of total data variance that is explained by the principal components of positive polarity 3D OrbiSIMS datasets. b) PC3 and PC4 scores.

Negative polarity

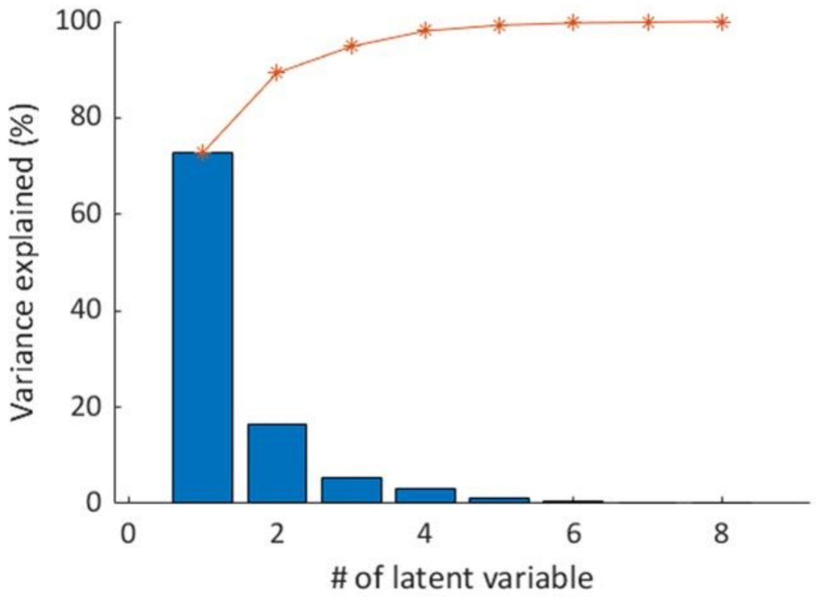

Figure S7. A bar chart showing the proportion of total data variance that is explained by the principal components of negative polarity 3D OrbiSIMS datasets.

Scores of PC1 and PC2

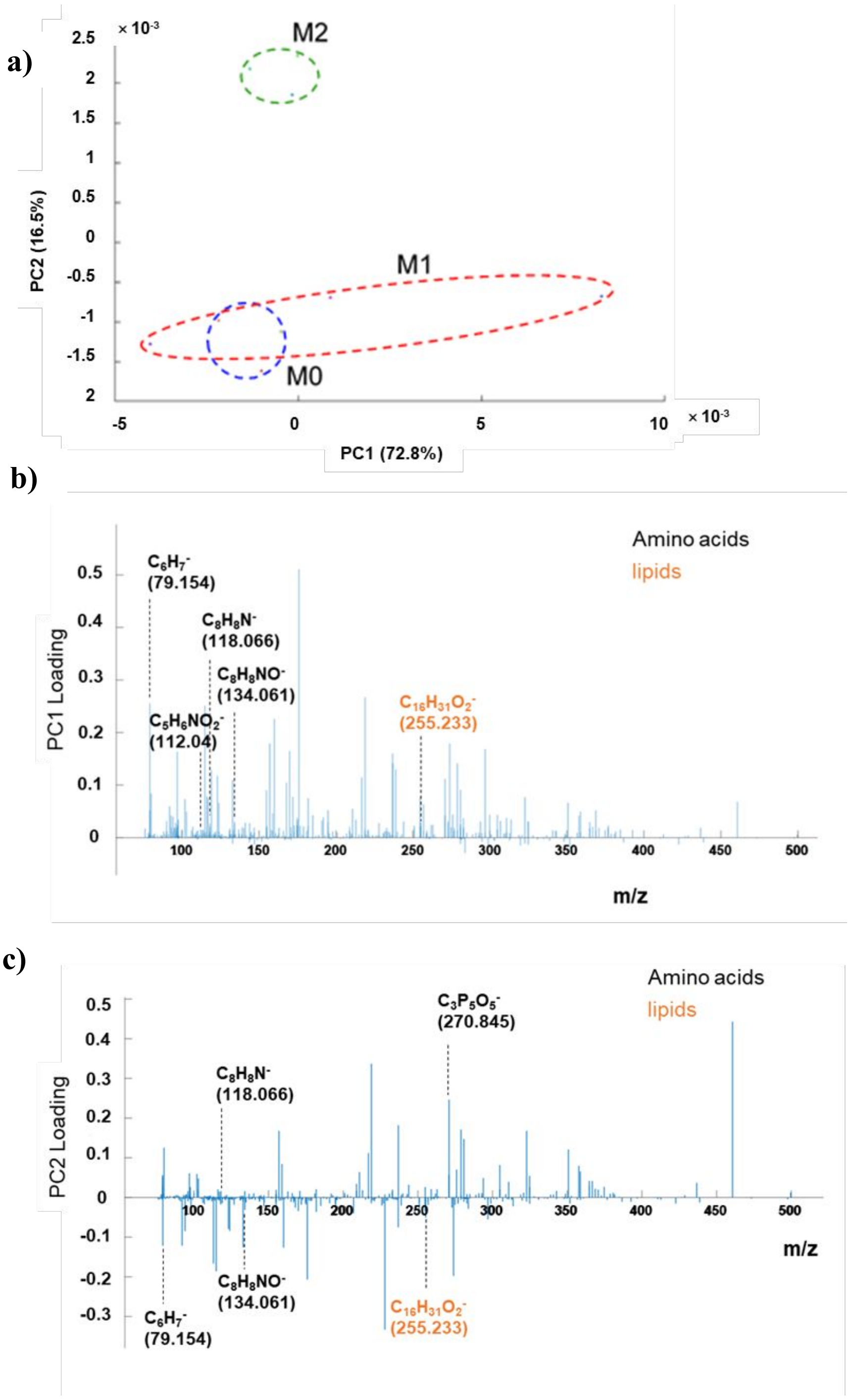

Figure S8. Principal component analysis on 3D OrbiSIMS negative polarity datasets. a) Scores of the first two principal components (PC). b) Loadings of PC1 and c) Loading of PC2. We attributed the $C_{16}H_{31}O_2^-$ as a larger lipid fragment due to its structural similarity to the larger lipid fragments, however we note it may also arise from fatty acids too.

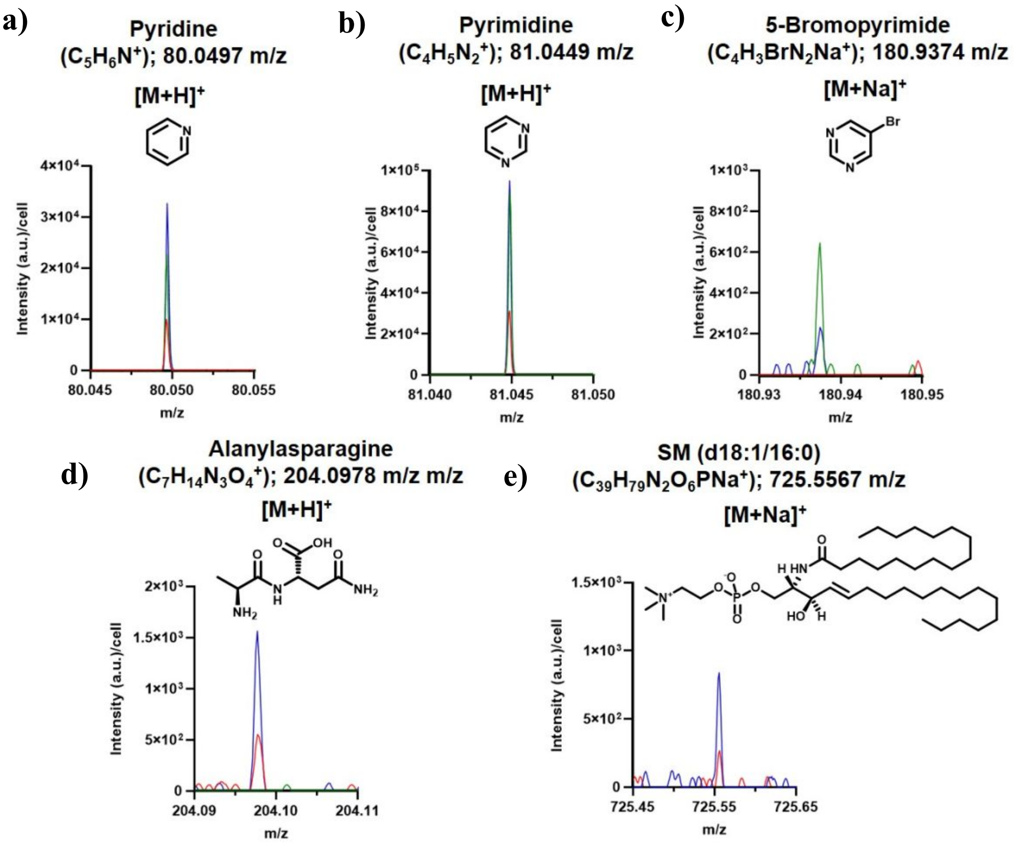

Figure S9. 3D OrbiSIMS spectrum of metabolite from a single cell macrophage each cell type in positive polarity. a) Pyridine $[M+H]^+$ at m/z 80.0497, b) Pyrimidine $[M+H]^+$ at m/z 81.0449, c) 5-bromopyridine $[M+Na]^+$ at m/z 180.9374, d) Alanylsparagine $[M+H]^+$ at m/z 204.0978, e) SM(d18/16:0) $[M+Na]^+$ at m/z 725.5567.
